# One-Page Patient Fact Sheets for Low Back Pain in Primary Care: A Randomized Clinical Trial

**DOI:** 10.1001/jamanetworkopen.2025.23352

**Published:** 2025-07-17

**Authors:** Christian Longtin, Jeremy R. Chang, Jolyn Hersch, Sweekriti Sharma, Michael C. Ferraro, Rodrigo R. N. Rizzo, Jennifer McBride, Arnold Y. L. Wong, Chris G. Maher, Michelle Guppy, James H. McAuley, Adrian C. Traeger

**Affiliations:** 1 School of Rehabilitation, University of Sherbrooke, Sherbrooke, Quebec, Canada; 2 Institute for Musculoskeletal Health, Sydney Local Health District, New South Wales, Australia; 3 Sydney School of Public Health, Faculty of Medicine and Health, The University of Sydney, Sydney, New South Wales, Australia; 4 Department of Rehabilitation Sciences, The Hong Kong Polytechnic University, Hong Kong SAR, China; 5 Centre for Pain IMPACT, Neuroscience Research Australia, Sydney, Australia; 6 School of Health Sciences, Faculty of Medicine and Health, University of New South Wales, Sydney, Australia; 7 Maridulu Budyari Gumal, the Sydney Partnership for Health, Education, Research and Enterprise (SPHERE) Consumer Reference Group, Sydney, Australia; 8 Research Institute for Smart Ageing, The Hong Kong Polytechnic University, Hong Kong SAR, China; 9 School of Rural Medicine, University of New England, Armidale, Australia

## Abstract

**Importance:**

Supporting informed decision-making is a fundamental aspect of primary care for low back pain. Two alternative approaches are providing fact sheets that list information about managing back pain or fact sheets that give advice on how to self-manage back pain, the comparative effects of which are unknown.

**Objective:**

To determine the effects of 1-page fact sheets on patient-reported preparedness for shared decision-making among people with low back pain.

**Design, Setting, and Participants:**

This 2-group parallel randomized clinical trial recruited 1080 adults from April 1, 2023, to April 30, 2024, who had recently seen their physician for uncomplicated low back pain. Participants were randomized to receive 1 of 2 one-page fact sheets. Outcomes were collected immediately after reading the fact sheet.

**Interventions:**

The information sheet was a 1-page fact sheet from a reputable medical journal and listed guideline-recommended management options. The advice sheet was a 1-page fact sheet from a recent clinical care standard and provided advice on how to self-manage low back pain.

**Main Outcomes and Measures:**

The primary outcome was patient-reported preparedness for decision-making, measured by the 100-point Preparation for Decision Making scale (where higher scores indicated higher preparedness for decision-making). Secondary outcomes included intentions for guideline-recommended interventions (staying active, physical therapies, and heat application) and nonrecommended interventions (imaging and opioid medicines). Analysis was performed on an intent-to-treat basis.

**Results:**

The total study sample included 1080 patients (mean [SD] age, 51.9 [14.7] years; 764 women [70.7%]). After reading the fact sheet, mean (SD) scores for preparation for decision-making were 57.6 (26.5) in the information sheet group and 52.9 (26.2) in the advice sheet group (adjusted mean difference, 4.7 points; 95% CI, 1.0-8.5 points; *P* = .01). A larger effect was observed among participants with chronic low back pain (mean [SD], 58.3 [28.9] points with information sheet vs 51.9 [28.4] points with advice sheet; adjusted mean difference, 6.4 points; 95% CI, 1.7-11.0 points; *P* *=* .007) compared with those with acute low back pain (mean [SD], 57.8 [26.0] points with information sheet vs 56.6 [29.2] points with advice sheet; adjusted mean difference, 1.2 points; 95% CI, −4.9 to 7.4 points; *P* = .70). There were no significant between-group differences in intentions to use guideline-recommended or nonrecommended interventions.

**Conclusion and Relevance:**

In this randomized clinical trial among people who had recently seen their physician for uncomplicated low back pain, an information sheet listing management options was more effective than an advice sheet at preparing patients for shared decision-making. It is uncertain whether this effect is meaningful.

**Trial Registration:**

http://anzctr.org.au Identifier: ACTRN12623000603617

## Key Points

**Question:**

What is the comparative effectiveness of 2 different fact sheets in preparing patients with low back pain for shared decision-making?

**Findings:**

In this randomized clinical trial of 1080 people who had recently seen their physician for low back pain, a nondirective information sheet listing treatment options was superior to a directive advice sheet on measures of patient-reported preparedness for decision-making.

**Meaning:**

This study suggests that fact sheets listing treatment options may better support decision-making than a traditional advice sheet.

## Teaser

This randomized clinical trial compares the effects of 1-page fact sheets that adopt either an information-listing approach or a direct advice approach on management of low back pain to prepare patients for shared decision-making

## Introduction

Low back pain is the second most common reason for seeking care from a primary care physician.^1^ There is high-quality evidence that information and advice in primary care can reassure patients and reduce health care use.^2^ Providing patients with education about low back pain underpins clinical management and is recommended by all clinical guidelines.^3^ The recent National Institutes of Health Helping to End Addiction Long-term initiative report found that the most preferred education resource for people with pain conditions was a 1-page fact sheet from their physician.^4^ However, primary care physicians have reported a lack of high-quality resources, and the optimal approach to supporting patient decision-making is uncertain.^5^

Ideally, the information resources provided by physicians should support evidence-based care and prepare patients for a shared decision-making process. Shared decision-making can improve patient satisfaction and adherence,^6^ reduce low-value care,^7^ increase uptake of high-value care,^8^ and support patient-centered decisions.^8^ Written information resources have also been shown to enhance understanding of treatment options and reduce decisional uncertainty in primary care.^9^ However, few patients in primary care report receiving information^10^ or engaging in shared decisions.^11^ A critical first step toward patient-centered care is to ensure that resources, such as fact sheets, optimally prepare them to make decisions about their care.

Several factors could challenge a shared decision-making process in consultations for low back pain. Many patients believe that imaging is essential to identify the source of their pain and prefer nonrecommended passive treatments, such as medications or rest.^12,13,14^ Providing patients with education resources could help inform decisions and align preferences with guideline-recommended care. One approach is to provide an information sheet,^15^ which uses a nondirective approach to describe a broad range of guideline-recommended management options available. A contrasting approach is a fact sheet that focuses on advice about a small number of recommended self-management strategies.^16^ Both approaches are currently used in clinical practice, but the comparative effects on decision-making have not been investigated, to our knowledge.^17^

We aimed to determine the relative effects of 2 currently used, 1-page fact sheets on low back pain that adopt either an information-listing approach or a direct advice approach. Outcomes of interest were patient-reported preparedness for shared decision-making and health care–seeking intentions.

## Methods

### Setting and Design

This study was a randomized, 1:1, 2-arm, parallel group, open-label, superiority clinical trial. The trial protocol in Supplement 1 was approved by The University of Sydney Human Research Ethics Committee and registered in the Australian New Zealand Clinical Trials Registry (ACTRN12623000603617). The study followed the Consolidated Standards of Reporting Trials (CONSORT) reporting guideline.^18^ Data were collected from April 1, 2023, to April 30, 2024. All participants provided online informed consent prior to data collection.

Participants aged 18 years or older who had consulted a physician for uncomplicated low back pain at least once in the past 4 weeks were eligible. Potential participants were recruited via 2 methods, either via an SMS (short message service) invitation from their physician’s clinic after their consultation or via an online advertisement on a social media platform (Facebook, Twitter, and Instagram). Physicians were eligible and automatically enrolled if they used the physician software provided by our software partner HealthShare Digital and if their patient opted in to receive additional information about back pain.

We used 2 computer-generated randomization sequences that, depending on the recruitment pathway, were used to allocate participants to receive 1 of the 2 fact sheets. Randomization occurred after the participant’s consultation with the physician. For participants recruited from physician practices, we used a randomization process based on the timing of the patient’s visit. A concealed randomization sequence determined whether a given month was designated for the advice sheet or the information sheet. The physician’s consultation software (Medical Director or Best Practice, the 2 most common consultation software products in Australia) was equipped with a software add-on that adjusted access to the relevant fact sheet each month, which could then be distributed to all consenting patients via SMS on behalf of the practice. For participants recruited through an online advertisement, we used the randomization process embedded in the Qualtrics data collection software, where eligible participants were randomized to receive 1 of the 2 fact sheets. These randomization approaches minimized selection bias while allowing for practical implementation across recruitment settings. Block randomization was not used for either randomization sequence. We did not assess whether physicians were aware of which fact sheet their patients received. Researchers and physicians were not blinded to group allocation; however, because group allocation was automated, there was limited to no opportunity for physicians or researchers to influence a participant’s responses.

Eligible participants completed a consent form and provided demographic and clinical information (eg, age, sex, pain intensity, and duration). Outcomes on preparedness for shared decision-making, health care–seeking intentions, and acceptability of the allocated fact sheet were collected immediately after the patient read the fact sheet in a web browser.

### Interventions

The information sheet was the *JAMA Patient Page* published on July 20, 2021.^15^ It describes guideline-endorsed medical and nonmedical treatment options for low back pain, with only limited advice. The advice sheet was taken from an Australian clinical care standard published on September 22, 2022,^16^ and provides a limited set of options using language that directs patients on how best to self-manage low back pain.

### Outcomes

The primary outcome was preparedness for shared decision-making, measured using the 10-item Preparation for Decision Making (PrepDM) scale.^19^ The PrepDM scale was designed to evaluate patients’ perceptions of how effectively an educational resource prepared them to engage in discussions with their practitioner and to make informed health decisions. Items included questions such as “To what extent did this education material prepare you to make a better decision?”^19^ The PrepDM scale has discriminated between educational resources in randomized clinical trials^7,20^ and has demonstrated good psychometric properties (eg, construct validity and unidimensionality).^19^ The items were summed, based on scores on a 5-point response scale (from “Not at all” to “A great deal”), to obtain a total score on a scale of 0 to 100, where higher scores indicated higher preparedness for decision-making.

Secondary outcomes were intentions to seek health care for guideline-recommended interventions (staying active, physical therapies, and heat application) and nonrecommended interventions (imaging and opioid medicines). These intentions were measured using an ordinal scale consisting of 4 options: definitely take, likely to take, unsure, not likely to take, and definitely not take.^21^ Complete questions and response categories are provided in eTable 1 in Supplement 2. We also assessed acceptability of the fact sheet regarding its length, informativeness, balance in encouraging medical or nonmedical approaches, comprehensibility, and likelihood of recommending the resource to others. These were measured either as an ordinal scale or as categorical variables. Complete questions and response categories are provided in eTable 2 in Supplement 2.

### Statistical Analysis

A 2017 Cochrane review of trials comparing patient decision aids vs usual care found the mean effect on decision-making to be 12 points on a 100-point scale.^7^ We tested the comparative effectiveness of 2 active interventions, 1-page fact sheets, and nonformal decision aids, so we chose a priori an effect size that was approximately 50% of the expected effect size of a patient decision aid found in the Cochrane review. Assuming a small mean difference of 6 points on a 100-point scale (ie, 60 points in the decision support sheet group and 54 points in advice sheet group, with an SD of 26) with 90% power, we required a sample size of 790 for our primary analysis. Our primary analysis compared the mean PrepDM scores among participants randomized to the information sheet vs the advice sheet with 95% CIs. We estimated the effect of the information sheets on the primary outcome using a multivariable linear regression model, with intent-to-treat principles. The main analysis included all participants who received a fact sheet and provided complete responses to the PrepDM scale. All statistical tests were 2-sided. The standardized difference between groups was calculated using established methods,^22^ interpreted as small (Cohen *d* > 0.2), medium (Cohen *d* > 0.5), and large (Cohen *d* > 0.8) effect sizes.^23^ We adjusted for recruitment source (ie, recruited from the physician’s clinic or via the community) in the analyses. Sensitivity analyses examined if our results were robust to removing “speeders” (ie, people who spent little time [<120 seconds] reading the fact sheets) from the sample. We tested whether removing these participants from our analysis affected the results. We conducted planned subgroup analyses to assess if the effect of the fact sheets differed among patients with chronic (>3 months) vs acute (≤3 months) pain and among patients with higher pain intensity (>6 of 10 on a visual analogue scale) vs lower pain intensity (≤6 of 10 on a visual analogue scale). For each of the analyses, we added an interaction term (group × moderator) variable in the linear regression models. Post hoc pairwise comparisons were conducted using the Tukey honest significant difference test to compare mean values between groups for both primary and secondary analyses. For the secondary outcomes, we estimated effects using ordinal logistic regression models. As with the primary analysis, we adjusted for recruitment source. To account for multiple comparisons in the secondary analyses, we set the significance threshold at *P* < .01. Results are presented as adjusted odds ratios (AORs) and 95% CIs (eMethods in Supplement 2). Statistical analyses were performed using R, version 4.4.1 (R Project for Statistical Computing).^24^

## Results

**Figure 1.**
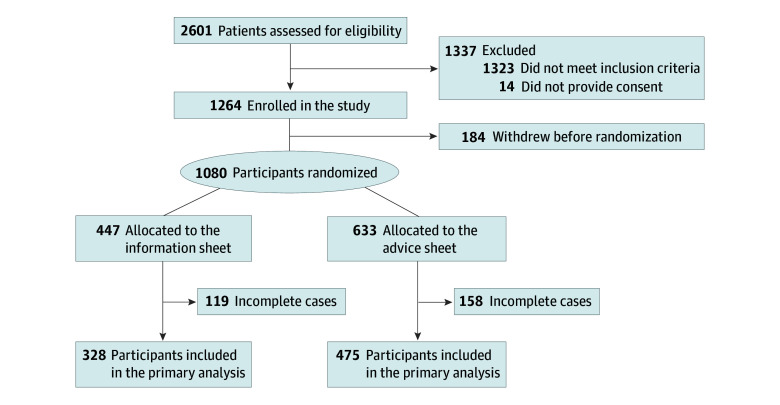
Flow of Participants Through the Study

The total study sample included 1080 patients (mean [SD] age, 51.9 [14.7] years; 764 women [70.7%] and 263 men [24.4]; Table) with a mean (SD) pain intensity of 6.8 (1.9). Between April 2023 and April 2024, 2601 individuals were assessed for eligibility (Figure 1). Overall, 1080 participants were randomized to receive 1 of the 2 fact sheets (information sheet, 447; advice sheet, 633), of whom 803 (74.4%) provided complete primary outcome data. The remainder (277 [25.6%]) were excluded because they provided only partial responses on the PrepDM scale; thus, a valid scores could not be computed. For the secondary outcomes, data are presented in eFigure 1 in Supplement 2.

**Table.** Characteristics of Participants

Characteristic	Overall (N = 1080)	Information sheet (n = 447)	Advice sheet (n = 633)	Standardized difference (95% CI)
Age, mean (SD), y	51.9 (14.7)	51.8 (14.7)	52.0 (14.8)	−0.01 (−0.13 to 0.11)
Sex, No./total No. (%)				
Female	764/1080 (70.7)	315/447 (70.5)	449/633 (70.9)	−0.01 (−0.13 to 0.11)
Male	263/1080 (24.4)	114/447 (25.5)	149/633 (23.5)	0.05 (−0.08 to 0.17)
Prefer not to say	53/1080 (4.9)	18/447 (4.0)	35/633 (5.5)	−0.07 (−0.19 to 0.05)
Recruitment source, No./total No. (%)				
Physician clinics	524/1080 (48.5)	191/447 (42.7)	333/633 (52.6)	−0.20 (−0.32 to −0.08)
Online advertisement	556/1080 (51.5)	256/447 (57.3)	300/633 (47.4)	0.20 (0.08 to 0.32)
Born in Australia, No./total No. (%)	736/1031 (71.4)	316/431 (73.3)	420/600 (70.0)	0.07 (−0.05 to 0.20)
Non-English language spoken at home, No./total No. (%)	182/1031 (17.7)	76/431 (17.6)	106/600 (17.7)	<−0.01 (−0.13 to 0.12)
First episode of low back pain, No./total No. (%)	162/1018 (15.9)	72/426 (16.9)	90/592 (15.2)	−0.05 (−0.08 to 0.17)
Pain intensity over the past week, mean (SD)^a^	6.8 (1.9)	6.7 (1.8)	6.8 (1.9)	−0.05 (−0.17 to 0.07)
Pain intensity categorized, No./total No. (%)^b^				
High pain	608/993 (61.2)	257/416 (61.8)	351/577 (60.8)	0.02 (−0.11 to 0.15)
Low pain	385/993 (38.8)	159/416 (38.2)	226/577 (39.2)	−0.02 (−0.15 to 0.11)
Pain duration, No./total No. (%)^c^				
Acute	337/1018 (33.1)	125/426 (29.3)	212/592 (35.8)	−0.14 (−0.26 to −0.02)
Chronic	681/1018 (66.9)	301/426 (70.7)	380/592 (64.2)	0.14 (0.02 to 0.26)

^a^ Pain intensity over the past week was assessed by a 0 to 10 scale (0 = no pain, 10 = worst pain imaginable).

^b^ Pain intensity was categorized as high pain intensity (>6 of 10 on visual analogue scale) vs low pain intensity (≤6 of 10 on visual analogue scale).

^c^ Pain duration was classified as chronic (>3 months) vs acute (≤3 months) pain.

Participants’ characteristics are summarized in the Table. The 2 intervention groups had similar characteristics. A slightly higher proportion of participants in the information sheet group had chronic low back pain compared with those in the advice sheet group (70.7% [301 of 426] vs 64.1% [380 of 592]; Cohen *d* = 0.14; 95% CI, 0.02-0.26). Approximately half of the participants (524 of 1080 [48.5%]) were recruited immediately after consultation with their physicians. Characteristics of completers and noncompleters were generally similar (eTable 3 in Supplement 2). Any between-group differences for completers vs noncompleters were below the threshold for a small effect size, with the exception that completers were less likely to speak a non-English language at home compared with noncompleters (13.8% [111 of 803] vs 27.6% [63 of 228]; Cohen *d* = −0.28; 95% CI, −0.43 to −0.13).

The primary analysis indicated that the information sheet was superior to the advice sheet on preparedness for shared decision-making; the mean (SD) PrepDM scores were 57.6 (26.5) in the information sheet group and 52.9 (26.2) in the advice sheet group (adjusted mean difference, 4.7 points; 95% CI, 1.0-8.5 points; Cohen *d* = 0.18; 95% CI, 0.04-0.32; *P* = .01) on a 100-point scale. The sensitivity analysis (eTable 4 in Supplement 2) supported the robustness of the results since the effect of the information sheets was similar with speeders removed (adjusted mean difference, 5.0 points; 95% CI, 1.3-8.7 points; *P* = .009).

The subgroup analyses (eTable 5 in Supplement 2) found that the information sheet had larger effects than the advice sheet among participants who had chronic low back pain (mean [SD], 58.3 [28.9] points with information sheet vs 51.9 [28.4] points with advice sheet; adjusted mean difference, 6.4 points; 95% CI, 1.7-11.0 points; *P* = .007) compared with those with acute low back pain (mean [SD], 57.8 [26.0] points with information sheet vs 56.6 [29.2] points with advice sheet; adjusted mean difference, 1.2 points; 95% CI, −4.9 to 7.4 points; *P* = .70). The subgroup analysis for pain intensity did not show statistically significant differences.

**Figure 2.**
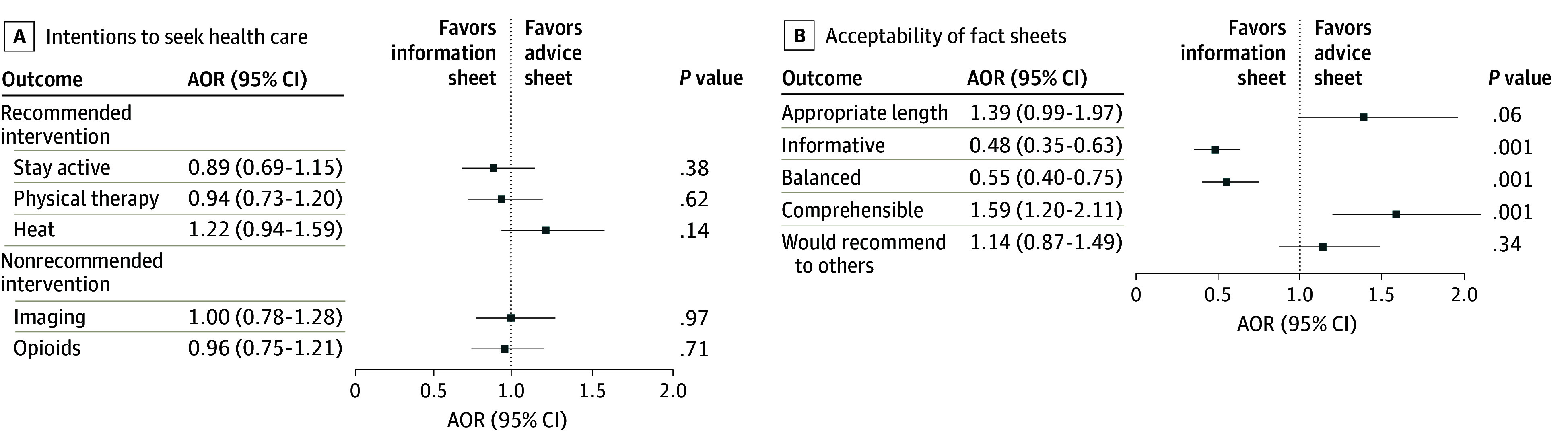
Effects of Information Sheets on Health Care–Seeking Intentions and Acceptability Measures AOR indicates adjusted odds ratio.

For the secondary outcomes, there were no significant between-group differences in intentions to use the 3 recommended interventions. Intentions to stay active (AOR, 0.89; 95% CI, 0.69-1.15; *P* = .38), use physical therapies (AOR, 0.94; 95% CI, 0.73-1.20; *P* = .62), and use heat therapy (AOR, 1.22; 95% CI, 0.94-1.59; *P* = .14) were similar between groups (Figure 2A). There were no differences in intentions to use nonrecommended interventions. Intentions to seek imaging investigations (AOR, 1.00; 95% CI, 0.78-1.28; *P* = .97) and to use opioids (AOR, 0.96; 95% CI, 0.75-1.21; *P* = .71) were similar between groups. The advice sheet was more likely to be considered comprehensible (AOR, 1.59; 95% CI, 1.20-2.11; *P* = .001) but less likely to be judged as informative (AOR, 0.48; 95% CI, 0.35-0.63; *P* = .001) and balanced (AOR, 0.55; 95% CI, 0.40-0.75; *P* = .001) compared with the information sheet (Figure 2B). No significant difference was observed between the 2 fact sheets in recommending the resource to others (AOR, 1.14; 95% CI, 0.87-01.49; *P* = .34).

## Discussion

An information sheet for patients, which focused on listing a range of management options rather than providing direct advice, was more effective than an advice sheet at supporting preparedness for decision-making, especially among patients with chronic low back pain. Neither information sheet showed superiority regarding intentions to use recommended or nonrecommended care. These findings challenge clinical guideline recommendations to focus on advice to self-manage; although effects were small, patients appeared to be more prepared to discuss options with their physicians and make future decisions when simply provided with evidence-based information on the range of treatment options.

There are several implications of our findings for future research and practice. The lack of differences in intentions for future health care suggests that providing direct advice alone might not achieve the desired changes to behavior. If alignment of patient preferences with clinical guidelines is the primary objective of an education resource, more persuasive and targeted approaches may be required. For example, describing the potential harms of unnecessary imaging and actively promoting opioid deprescribing could influence intentions.^25,26^

Participants exhibited significantly different evaluations of the 2 approaches regarding informativeness, balance, and comprehensibility. This finding indicates a potential trade-off between informativeness and balance, which were superior with the information listing approach, and perceptions of comprehensibility and length, which were superior with the advice approach. Future research could assess comprehension more directly, for example, through think-aloud interviews and qualitative methods.

Although the fact sheets differed most markedly in their approach to providing treatment options (information listing vs advice), because these were materials currently used in practice, it is possible that the observed effects were due to other differences between the resources. For example, there were some differences in visual aspects, type of content (eg, coverage of medical vs nonmedical care options), word count, language, and readability. We used the Sydney Health Literacy Lab Editor^27^ to assess readability of both sheets and found a higher readability score for the advice sheet compared with the information sheet.^28^ Ensuring resources not only support decision making but are accessible and easy to understand for most patients is a critical future consideration. Health literacy editors could enhance the effect of patient fact sheets developed in the future.^28,29^

The greater effect of the information sheet among patients with chronic low back pain may be due to these patients valuing content on a broader range of treatment options. The information sheet also offered a stepped-care approach, which lists alternative strategies if the initial approach to management is unsuccessful.^30^ Many people with chronic low back pain are already actively engaged in self-managing their pain, which could help explain the inferiority of the advice sheet in this group.^31^ In addition, it may be worthwhile to include clear information on both the benefits and risks of treatment options in patient fact sheets. This information could further support informed decision-making, as patients often overestimate the benefits and underestimate the harms of medical interventions.^32^ There was also a trend toward a larger between-group effect among people with low pain vs high pain (eTable 5 in Supplement 2). Future research is needed to examine the elements that contribute to the effectiveness of the information listing approach, especially for people with chronic low back pain, and the potential moderating influence of pain intensity.

### Strengths and Limitations

This trial has several strengths in comparison with previous research. To our knowledge, it is the first trial to compare the effects of 2 very brief interventions on decision-making about low back pain management in clinical practice.^33^ We conducted the trial among a large sample of patients compared with previous trials of patient education that had sample sizes ranging from 12 to 317 individuals and rarely measured decision-making outcomes.^17,34^ A 2024 Cochrane review located no trials of decision aids for people with uncomplicated back pain attending a primary care physician office visit.^35^ One pilot trial among 148 patients presenting to physiotherapists in the UK found that a decision support intervention had potential to worsen functional outcomes compared with usual care, but there was considerable uncertainty due to small sample size.^36^ Another trial conducted in the sickness insurance setting in Germany found that a tailored decision aid could have a 12-point effect on preparedness for decision-making compared with usual care, but more than 90% of participants were lost to follow-up, introducing substantial risk of attrition bias.^37^ The effect size observed in the present study (approximately 5 points on a 100-point scale) is small but should be considered in context. The mean effect size of patient decision aids compared with usual care on measures of knowledge was 12 points (95% CI, 10.6-13.2 points; 107 studies, 25 492 participants).^7^ This finding suggests that even a comprehensive patient decision aid is likely to have small effects. It is therefore arguable that when comparing 2 similar patient resources, such as a 1-page fact sheet, a trial should be powered to detect even small differences. To gather a large amount of data relevant to clinical care, we used innovative methods to identify and enroll participants directly from primary care via physician software and recruited a separate sample of participants from the community who were seeking care from a physician. The intervention was implemented without additional researcher oversight during clinical consultations, and the trial design sought to reflect the conditions under which such fact sheets would realistically be delivered. We consider this a key strength of the study. Effects were observed in the context of primary care, in which patients could be receiving multiple, potentially conflicting, sources of information at any given time.

This study also has some limitations. First, it is possible that some participants did not read the fact sheet completely. Many patients in clinical practice might not fully engage with education resources. However, our analysis accounted for participants who spent very little time reading the fact sheet. Second, we only measured preparedness for shared decision-making on 1 occasion immediately after reading the fact sheet, the effect was small, and the clinical meaningfulness was uncertain. However, given that we were comparing 2 currently used and readily available 1-page fact sheets, even small effects could be meaningful in this context. There is no cost for physicians to achieve small benefits in patient preparedness for decision-making. Third, we included participants irrespective of whether they had 1 or several consultations for back pain in the past 4 weeks. It is possible that the number of consultations could influence results if this factor was not evenly distributed between groups. Fourth, we did not collect other clinically important outcome measures, such as back pain–related disability or subsequent health care use. The impact of the effects observed on the PrepDM on these factors is therefore unclear.

## Conclusions

In this randomized clinical trial among people who had recently seen their physician for uncomplicated low back pain, an information sheet listing management options was more effective than an advice sheet at preparing patients for shared decision-making. It is uncertain whether this effect is meaningful.

## Notes

Supplement 1.
Trial Protocol and Statistical Analysis Plan

Supplement 2.
eMethods.
**eTable 1.** The Intentions to Seek Healthcare **eTable 2.** Acceptability of the Fact Sheet **eTable 3.** Characteristics of Completers and Non-completers **eTable 4.** Results of the Sensitivity Analysis Excluding “Speeders” **eTable 5.** Results of the Subgroup Analyses **eFigure.** Flow of Participants for Secondary Outcomes

Supplement 3.
Data Sharing Statement

## References

[1] Deyo RA, Weinstein JN. Low back pain. N Engl J Med. 2001;344(5):363-370. doi:10.1056/NEJM200102013440508 11172169

[2] Traeger AC, Hübscher M, Henschke N, Moseley GL, Lee H, McAuley JH. Effect of primary care–based education on reassurance in patients with acute low back pain: systematic review and meta-analysis. JAMA Intern Med. 2015;175(5):733-743. doi:10.1001/jamainternmed.2015.0217 25799308

[3] Oliveira CB, Maher CG, Pinto RZ, . Clinical practice guidelines for the management of non-specific low back pain in primary care: an updated overview. Eur Spine J. 2018;27(11):2791-2803. doi:10.1007/s00586-018-5673-2 29971708

[4] NIH HEAL Initiative: 2023 Annual Report. National Institutes of Health. February 16, 2023. Accessed June 11, 2025. https://heal.nih.gov/files/2023-08/2023-nih-heal-annual-report.pdf

[5] Slade SC, Kent P, Patel S, Bucknall T, Buchbinder R. Barriers to primary care clinician adherence to clinical guidelines for the management of low back pain: a systematic review and metasynthesis of qualitative studies. Clin J Pain. 2016;32(9):800-816. doi:10.1097/AJP.0000000000000324 26710217

[6] Hoffmann T, Bakhit M, Michaleff Z. Shared decision making and physical therapy: what, when, how, and why? Braz J Phys Ther. 2022;26(1):100382. doi:10.1016/j.bjpt.2021.100382 35063699 PMC8784295

[7] Stacey D, Légaré F, Lewis K, . Decision aids for people facing health treatment or screening decisions. Cochrane Database Syst Rev. 2017;4(4):CD001431. doi:10.1002/14651858.CD001431.pub5 28402085 PMC6478132

[8] Sepucha K, Bedair H, Yu L, . Decision support strategies for hip and knee osteoarthritis: less is more: a randomized comparative effectiveness trial (DECIDE-OA study). J Bone Joint Surg Am. 2019;101(18):1645-1653. doi:10.2106/JBJS.19.00004 31567801 PMC6887636

[9] Bowen E, Nayfe R, Milburn N, . Do decision aids benefit patients with chronic musculoskeletal pain? a systematic review. Pain Med. 2020;21(5):951-969. doi:10.1093/pm/pnz280 31880805 PMC7209464

[10] Verbeek J, Sengers MJ, Riemens L, Haafkens J. Patient expectations of treatment for back pain: a systematic review of qualitative and quantitative studies. Spine (Phila Pa 1976). 2004;29(20):2309-2318. doi:10.1097/01.brs.0000142007.38256.7f 15480147

[11] Davidson KW, Mangione CM, Barry MJ, ; US Preventive Services Task Force. Collaboration and shared decision-making between patients and clinicians in preventive health care decisions and US Preventive Services Task Force recommendations. JAMA. 2022;327(12):1171-1176. doi:10.1001/jama.2022.3267 35315879

[12] Almeida M, Saragiotto B, Richards B, Maher CG. Primary care management of non-specific low back pain: key messages from recent clinical guidelines. Med J Aust. 2018;208(6):272-275. doi:10.5694/mja17.01152 29614943

[13] Sharma S, Traeger AC, Reed B, . Clinician and patient beliefs about diagnostic imaging for low back pain: a systematic qualitative evidence synthesis. BMJ Open. 2020;10(8):e037820. doi:10.1136/bmjopen-2020-037820 32830105 PMC7451538

[14] French SD, Nielsen M, Hall L, . Essential key messages about diagnosis, imaging, and self-care for people with low back pain: a modified Delphi study of consumer and expert opinions. Pain. 2019;160(12):2787-2797. doi:10.1097/j.pain.0000000000001663 31356451

[15] Traeger AC, Qaseem A, McAuley JH. Low back pain. JAMA. 2021;326(3):286. doi:10.1001/jama.2020.19715 34283182

[16] How to manage your low back pain—information for patients. Australian Commission on Safety and Quality in Health Care. September 22, 2022. Accessed June 11, 2025. https://www.safetyandquality.gov.au/publications-and-resources/resource-library/how-manage-your-low-back-pain-information-patients

[17] Jones CM, Shaheed CA, Ferreira GE, Kharel P, Christine Lin CW, Maher CG. Advice and education provide small short-term improvements in pain and disability in people with non-specific spinal pain: a systematic review. J Physiother. 2021;67(4):263-270. doi:10.1016/j.jphys.2021.08.014 34518145

[18] Schulz KF, Altman DG, Moher D; CONSORT Group. CONSORT 2010 Statement: updated guidelines for reporting parallel group randomised trials. BMC Med. 2010;8(18):18. doi:10.1186/1741-7015-8-18 20619135

[19] Bennett C, Graham ID, Kristjansson E, Kearing SA, Clay KF, O’Connor AM. Validation of a preparation for decision making scale. Patient Educ Couns. 2010;78(1):130-133. doi:10.1016/j.pec.2009.05.012 19560303

[20] Muscat DM, Thompson R, Cvejic E, . Randomized trial of the Choosing Wisely consumer questions and a shared decision-making video intervention on decision-making outcomes. Med Decis Making. 2023;43(6):642-655. doi:10.1177/0272989X231184461 37403779 PMC10422858

[21] Hersch J, Barratt A, Jansen J, . The effect of information about overdetection of breast cancer on women’s decision-making about mammography screening: study protocol for a randomised controlled trial. BMJ Open. 2014;4(5):e004990. doi:10.1136/bmjopen-2014-004990 24833692 PMC4025472

[22] Yang D, Dalton JE. A unified approach to measuring the effect size between two groups using SAS: paper 335-2012. Presented at: SAS Global Forum 2012; April 22-25, 2012; Orlando, Florida.

[23] Cohen J. Statistical Power Analysis for the Behavioral Sciences. 2nd ed. Lawrence Erlbaum Associates; 1988.

[24] R Core Team. R: A Language and Environment for Statistical Computing. R Foundation for Statistical Computing. Published online 2024. Accessed May 22, 2025. https://www.r-project.org/

[25] Sharma S, Traeger AC, O’Keeffe M, . Effect of information format on intentions and beliefs regarding diagnostic imaging for non-specific low back pain: a randomised controlled trial in members of the public. Patient Educ Couns. 2021;104(3):595-602. doi:10.1016/j.pec.2020.08.021 32854984

[26] Jones KF, Stolzmann K, Wormwood J, . Patient-directed education to promote deprescribing: a nonrandomized clinical trial. JAMA Intern Med. 2024;184(11):1339-1346. doi:10.1001/jamainternmed.2024.4739 39312257 PMC11420822

[27] Health Literacy Editor. Sydney Health Literacy Lab. Accessed June 6, 2025. https://shell.techlab.works/

[28] Ayre J, Bonner C, Muscat DM, . Multiple automated health literacy assessments of written health information: development of the SHeLL (Sydney Health Literacy Lab) Health Literacy Editor v1. JMIR Form Res. 2023;7:e40645. doi:10.2196/40645 36787164 PMC9975914

[29] Ayre J, Muscat DM, Mac O, . Helping patient educators meet health literacy needs: end-user testing and iterative development of an innovative health literacy editing tool. PEC Innov. 2023;2:100162. doi:10.1016/j.pecinn.2023.100162 37384149 PMC10294045

[30] Lim YZ, Chou L, Au RT, . People with low back pain want clear, consistent and personalised information on prognosis, treatment options and self-management strategies: a systematic review. J Physiother. 2019;65(3):124-135. doi:10.1016/j.jphys.2019.05.010 31227280

[31] Du S, Hu L, Dong J, . Self-management program for chronic low back pain: a systematic review and meta-analysis. Patient Educ Couns. 2017;100(1):37-49. doi:10.1016/j.pec.2016.07.029 27554077

[32] Hoffmann TC, Del Mar C. Patients’ expectations of the benefits and harms of treatments, screening, and tests: a systematic review. JAMA Intern Med. 2015;175(2):274-286. doi:10.1001/jamainternmed.2014.6016 25531451

[33] Kang Y, Trewern L, Jackman J, Irani Nee Soni A, McCartney D. Chronic pain: supported self-management. BMJ. 2024;384:e072362. doi:10.1136/bmj-2022-072362 38167273

[34] Traeger AC, Lee H, Hübscher M, . Effect of intensive patient education vs placebo patient education on outcomes in patients with acute low back pain: a randomized clinical trial. JAMA Neurol. 2019;76(2):161-169. doi:10.1001/jamaneurol.2018.3376 30398542 PMC6440280

[35] Stacey D, Lewis KB, Smith M, . Decision aids for people facing health treatment or screening decisions. Cochrane Database Syst Rev. 2024;1(1):CD001431. 38284415 10.1002/14651858.CD001431.pub6 PMC10823577

[36] Patel S, Ngunjiri A, Hee SW, . Primum non nocere: shared informed decision making in low back pain—a pilot cluster randomised trial. BMC Musculoskelet Disord. 2014;15:282. doi:10.1186/1471-2474-15-282 25146587 PMC4247192

[37] Simon D, Kriston L, von Wolff A, . Effectiveness of a web-based, individually tailored decision aid for depression or acute low back pain: a randomized controlled trial. Patient Educ Couns. 2012;87(3):360-368. doi:10.1016/j.pec.2011.10.009 22154867

