# Supplementary material for: One-Page Patient Fact Sheets for Low Back Pain in Primary Care: A Randomized Clinical Trial
Source: JAMA Netw Open. 2025 Jul 17;8(7):e2523352. doi: 10.1001/jamanetworkopen.2025.23352 (PMC12272283; doi:10.1001/jamanetworkopen.2025.23352)
Supplement: Supplement 1. — Trial Protocol and Statistical Analysis Plan [file jamanetwopen-e2523352-s001.pdf]

Supplement Trial Protocol

This supplementary file includes the original study protocol, details of any amendments, and the trial registration record on the Australian New Zealand Clinical Trials Registry (ACTRN12623000603617).

TITLE
Back pain fact sheets and decisions about future healthcare

Investigators
Adrian C Traeger (Senior Research Fellow, The University of Sydney)
Swee Sharma (Research Fellow, The University of Sydney)
Christian Longtin (Visiting Research Fellow, the University of Sydney)

BACKGROUND
Patient education improves outcomes for people with low back pain. There is high quality evidence that written or verbal patient education can reassure patients and reduce subsequent healthcare utilisation in the short- and long-term.[1] It is the most common recommendation in international clinical guidelines for back pain. Yet primary care clinicians globally appear to underuse patient education.[2]

Easily accessed, evidence-based fact sheets, that can be printed or emailed to patients, could be a simple option to improve care for low back pain.[3] A Cochrane review found patient-mediated interventions, including access to written patient materials, may improve the appropriateness of care for mixed conditions, though the evidence was uncertain.[4] Fact sheets that help patients prepare for decision-making about their treatment options could encourage shared decision-making and better care.[5]

Digital health companies now provide consultation software add-ons that provide access to fact sheets during a consultation. SMS systems allow clinicians to routinely send fact sheets following a consultation. Two new fact sheets being implemented using these approaches in Australia in 2023 are the *JAMA Patient Page* (JAMA fact sheet) and the *Australian Commission for Healthcare Safety and Quality Information for Patients Fact Sheet* (ACHSQ fact sheet). Both resources are evidence-based but differ in their focus. A key difference is that the JAMA fact sheet focuses on listing evidence-based treatment options, whereas the ACHSQ fact sheet explains how a person can self-manage back pain.

There is no rigorous evidence on the effects of either of these new resources among people seeking care for low back pain. It is uncertain whether a fact sheet resource that focuses more on listing medical management options (JAMA fact sheet), can increase a patient's preparedness for decision-making, compared with a resource that focuses on self-management.

High-quality primary care-based studies on fact sheets and other patient information materials require an adequate response rate. Identifying methods to boost recruitment, such as different survey advertising methods (eg, reimbursement incentives and appeals to

---

[1] Traeger AC, Hübscher M, Henschke N, et al. JAMA Intern Med. 2015 May;175(5):733-43
[2] Traeger AC, et al. Bull World Health Organ. 2019:97(6):423-433.
[3] Hébert ET, Caughy MO, Shuval K. Br J Sports Med. 2012 Jul;46(9):625-31.
[4] Fønhus et al. Cochrane Database Syst Rev. 2018 Sep 11;9(9):CD012472.
[5] Bennett C, Graham ID, Kristjansson E, et al. Patient Educ Couns. 2010 Jan;78(1):130-3.
[6] Edwards PJ, Clarke IR, Diguiseppi C, et al. Cochrane Database Syst Rev. 2009 Jul;3.

social goods) could optimise research in electronic and postal questionnaires.[6] It is unclear which strategies can improve the recruitment of patients with back pain attending primary care who have received a fact sheet.

AIMS

Our primary aim is to compare preparation for decision-making among people seeking care for low back pain who are given one of two fact sheets. Secondary aims are to understand recruitment processes and engagement with research fact sheets, examine management intentions, feelings of reassurance about serious pathology and the acceptability of the fact sheets to people seeking care for low back pain.

METHODS

Consumer involvement

Our consumer partner CI McBride explained to the research team the value of receiving high-quality information about low back pain from one's GP. CI McBride helped us design our patient survey and suggested we document whether patients recalled their GP explaining the content of the fact sheet.

Design, participants, and setting

This will be a survey of people receiving one of the two fact sheets. Around 790 patients who have recently seen their GP for back pain will be surveyed. Participants could receive a fact sheet via one of three methods. The first method is directly from their GP who prints and/or emails the sheet to their patient (GP-led access, Figure 1). The GP determines which sheet they will provide and so receipt of the JAMA or ACHSQ fact sheet is non-random.

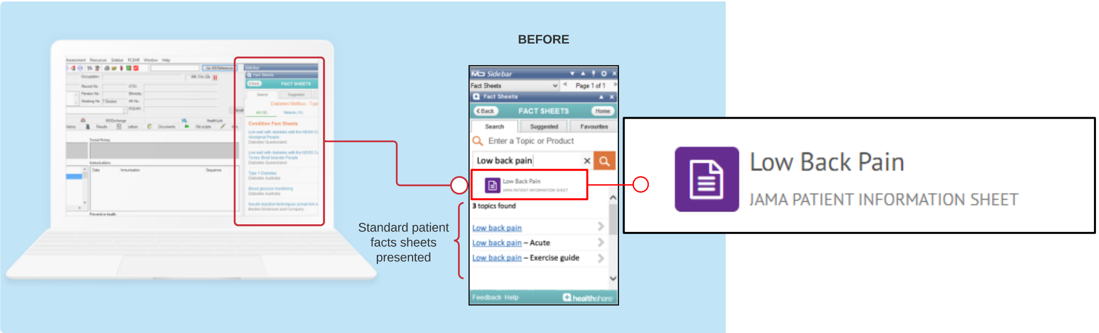

Figure 1. GP-led access to back pain fact sheet. Appearance of the HealthShare sidebar where GPs can access fact sheets that are relevant to what is typed into consultation software. A clinician can print or email the sheet to their patient as they wish.

The second method is patient-led access to a back pain fact sheet via an SMS system called BetterConsult. BetterConsult invites a patient to receive a back pain fact sheet after they see their GP (Figure 2). Whether the patient receives the JAMA or ACHSQ fact sheet is determined randomly by the fact sheet distributor, HealthShare. Patients can view the sheet on their smartphone or print the sheet out and their GP will be notified. GPs can open and

discuss the fact sheet with their patient during the consult if they wish. Twenty-four hours after their consultation with the GP, HealthShare sends patients an SMS containing a link to the fact sheet.

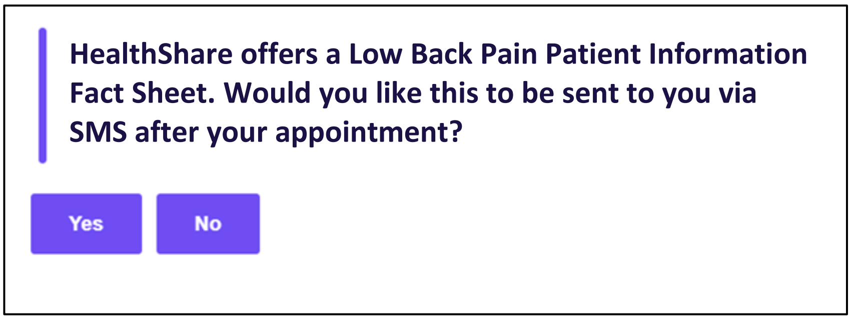

Figure 2. Patient-led access to back pain fact sheet. Patient opts to receive a fact sheet via SMS when they complete their details in a pre-consultation tool (BetterConsult).

The third method is via an advertisement on social media (Facebook, Twitter, and Instagram). The advertisement will invite people who have recently seen their GP for back pain to view a fact sheet and complete a short survey (See Appendix A).

The nature of the first two access methods means that we can make a randomised comparison of the survey responses to the two fact sheets delivered via patient-led access, but not those delivered by the GP-led access, ie in the consult by print or email. For people accessing the fact sheet via the third method (social media), the exposures will be randomised using Qualtrics software hosted by The University of Sydney. Data from patients receiving a fact sheet using the three methods (GP-led access vs Patient-led access vs Social Media) will be analysed separately in a sensitivity analysis.

Procedure

Participants will be people >18 years visiting their GP for back pain who receive a back pain fact sheet and who choose to complete the online survey. Patients who access the survey link and agree to participate in the study will provide clinical and demographic details via an online form (age, sex, ethnicity, duration of back pain, pain intensity). No data from a participant's medical record will be used in this study.

COPYRIGHTED MATERIAL REDACTED

Figure 3. Potential participants can access the survey via a QR code on printed/emailed sheet or via clickable link on the fact sheet page or via social media advertisements.

Outcomes

The **primary outcome** of this study is preparation for decision-making, measured using the Preparation for Decision Making (PrepDM) scale.[6] PrepDM has 10-items that address concepts of preparedness for decision-making. It has successfully discriminated between different educational resources in randomised trials and is valid and reliable.

Secondary outcomes

- Management intentions

  We adapted items from Hersch et al.[7] for decisions about care for low back pain:

  - At the moment, which of the following best describes your intentions about **having a scan (x-ray, CT, MRI)** for your low back pain?

    Response options:

    - I definitely will have a scan
    - I am likely to have a scan
    - I am unsure

---

[6] Bennett C, Graham ID, Kristjansson E, et al. Patient Educ Couns. 2010 Jan;78(1):130-3

[7] Hersch J, et al. BMJ Open. 2014 May 15;4(5):e004990.

    - I am NOT likely to have a scan
    - I definitely will NOT have a scan

- At the moment, which of the following best describes your intentions about **taking opioids (strong prescription pain medicines e.g. oxycodone, codeine, tramadol)** for your low back pain?
    - I definitely will take opioids
    - I am likely to take opioids
    - I am unsure
    - I am NOT likely to take opioids
    - I definitely will NOT take opioids

- At the moment, which of the following best describes your intentions about **using heat (e.g. heat packs, hot water bottle)** for your low back pain?
    - I definitely will use heat
    - I am likely to use heat
    - I am unsure
    - I am NOT likely to use heat
    - I definitely will NOT use heat

- At the moment, which of the following best describes your intentions about **seeking physical therapy on your back (e.g. massage or spinal manipulation)**?
    - I definitely will seek physical therapy
    - I am likely to seek physical therapy
    - I am unsure
    - I am NOT likely to seek physical therapy
    - I definitely will NOT seek physical therapy

- At the moment, which of the following best describes your intentions about **staying active** while you have low back pain?
    - I definitely will stay active
    - I am likely to stay active
    - I am unsure
    - I am NOT likely to stay active
    - I definitely will NOT stay active

Participants who report being definitely/likely to seek imaging or opioids for their low back pain will be classified as having “intentions for medical care”.

- Reassurance

- How reassured are you that there is no serious condition causing your low back pain? (from Traeger et al)[8] 0 - not reassured at all; 10 - completely reassured

- Acceptability
  - Did you read the fact sheet all the way through?

    If Yes - how many minutes did you spend reading the fact sheet?
    [_______ min] .
    If No - How much of the fact sheet did you read?
    [options: most / some / a little]

  - How would you rate the length of the fact sheet?

    [options: much too short / a little too short / just about right / a little too long / much too long]

  - How much of the information in the fact sheet was new to you?

    [options: all / most / some / none]

    The fact sheet talked about different ways to manage back pain. It mentioned **medical care (pain medicines, imaging, surgery)** and **non-medical care (massage, heat, staying active)** for back pain. Did you feel the fact sheet was: [options: leaning very much towards medical care/ leaning a bit towards medical care/Balanced/ Leaning a bit towards non-medical care/Leaning very much towards non-medical care]

  - How strongly do you agree or disagree with each of the following statements.

    [options: strongly agree / agree / neither agree nor disagree / disagree / strongly disagree]

    - I found the information in the fact sheet clear and easy to understand.
    - I would recommend this fact sheet to other people who are considering their options to treat low back pain.

- Fidelity
  - How did you receive the fact sheet
    - From my GP
    - Via SMS from my GP practice
    - Social media
    - Other [please describe]

- Implementation

[8] Traeger AC et al. JAMA Neurol. 2019 Feb 1;76(2):161-169.

- We will also conduct semi-structured interviews and or focus groups (depending on feasibility) with up to 20 clinicians and 20 patients (interview only) to identify barriers and facilitators to implementation of the fact sheets in primary care. We used the COM-B framework to create our initial interview guide (Appendix D) which will be updated iteratively as interviews/focus groups progress. For the clinician focus groups if feasible, we will conduct up to 3 focus groups with 5-6 GPs in each group.

- Recruitment rates
  - Proportion of people exposed to the survey advertisement who consent to participate

Nested study:

BACKGROUND: High-quality primary care-based research requires adequate response rates. A recent systematic review identified some promising strategies including monetary incentives.[9] However, most evaluations to date have focused on mail and telephone-based patient experience surveys.

AIM: assess the effect of different survey advertising methods using fact sheets embedded in consultation software (BetterConsult provided by the company HealthShare), on recruitment rate.

DESIGN: ABACAD replicated time series design where "A" are control periods and "B" and "C" are different behaviourally informed advertising strategies.

PARTICIPANTS: Patients who have recently seen their GP for back pain and have received back pain fact sheet. Participants could receive a fact sheet via one of two methods. The first method is directly from their GP who prints and/or emails the sheet to their patient. The GP determines which sheet they will provide and so receipt of the JAMA or ACHSQ fact sheet is non-random. The second method is patient-led access to a back pain fact sheet via an SMS system called BetterConsult. BetterConsult invites a patient to receive a back pain fact sheet after they see their GP. Whether the patient receives the JAMA or ACHSQ fact sheet is determined randomly by the fact sheet distributor, HealthShare. Patients can view the sheet on their smartphone or print the sheet out and their GP will be notified. GPs can open and discuss the fact sheet with their patient during the consult if they wish. Twenty-four hours after their consultation with the GP, HealthShare sends patients an SMS containing a link to the fact sheet.
Final sample size will be determined by monthly reach of study advertising.

EXPOSURES: Control Period A – Advertisement is a clickable link and QR code with the words "Have your say about this fact sheet" in a red box in the top right corner of the fact sheet. Intervention Period B – Advertisement is a clickable link that mentions a financial incentive to participate: "Have your say about this fact

[9] Price et al (2022) Med Care. 2022 Dec; 60(12): 910–918.

sheet. Complete a short survey **for your chance to win**" is written in a red box in the top right corner of the fact sheet. Intervention Period C – Advertisement is a clickable link with phrasing that appeals to social good: "**Help us improve fact sheets for people with back pain**." Intervention Period D – Advertisement is a clickable link with phrasing that appeals to both social good and provides financial incentive: "**Help us improve fact sheets for people with back pain**. Complete a short survey **for your chance to win**" in a red box in the top right corner of the fact sheet.

COPYRIGHTED MATERIAL REDACTED

COPYRIGHTED MATERIAL REDACTED

Intervention period B

COPYRIGHTED MATERIAL REDACTED

COPYRIGHTED MATERIAL REDACTED

Figure 4: Three different advertising strategies to increase response rate to a fact sheet-based survey.

OUTCOMES: Proportion of people exposed to the survey advertisement who consent to participate

SAMPLE SIZE: A previous study comparing recruitment rates of different online recruitment methods suggest 27% response rate.[9] To detect a difference in response rate of 5% (27% in control group, 32% in intervention group), with 80% power and 0.05 alpha, sample size required would be 1306 participants in each group (total n= 5224). The recruitment will stop at 5224 participants.

ANALYSIS: Descriptive analyses will be done to report the response rate for all participants according to trial period. We will compare the number and proportion of participants responding to messages.

ETHICAL ISSUES:
Consent
We are seeking a waiver of consent for participants of the nested study (ie people exposed to the advertisement) because it is impractical and because obtaining consent would create a greater burden to participants than the interventions themselves.

Monetary incentives
Fair distribution of benefits and risks of research is one of the key principles of ethical research. Participants invest their time to participate in research deserve

some benefit in return. We will provide this in the form of a ticket to a lucky draw to win one of 5 Myer vouchers valued at $100.

It is appropriate to provide such incentive to participants in research projects that are not risk rated as high risk, such as this project.[10] We will maintain the anonymity of responses, while having a mechanism to send participants the incentive and enter them into the draw at the same time. For this we will separate the data collection and the incentive mechanism by entering the data from the completed instrument and the incentive entry in completely separate tables without any relational link between the two tables.

In running the lucky draw, we will comply with the NSW lottery laws and draws will be administered and conducted by someone independent of the research team. We are using the same approach in another HREC approved project (ID 2023/ETH00472 – Southwest Sydney Local Health District HREC)

Exposures

Exposures will be two recently published, evidence-based resources for low back pain. The *JAMA Patient Page* (Available from: https://jamanetwork.com/journals/jama/fullarticle/2782184) was published on 20/7/21 and describes guideline-endorsed medical and non-medical management options for low back pain.
The *Australian Commission for Healthcare Safety and Quality (ACHSQ) Information for Patients Fact Sheet* (Available from: https://www.safetyandquality.gov.au/publications-and-resources/resource-library/how-manage-your-low-back-pain-information-patients) was published on 22/9/22 and describes how to self-manage low back pain.
Both factsheets have been implemented into routine practice via HealthShare consultation tools.

Statistical analysis

Our primary analysis will compare the PrepDM scores in people exposed to the JAMA fact sheet vs the ACHSQ fact sheet, immediately after they have read the sheet. Assuming a mean difference of 6 points on a 100-point scale (ie 60 points in JAMA fact sheet group, 54 points in ACHSQ group, SD of 26) with 90% power, we require a sample size of 790.[11] To account for anticipated uneven group size we will increase the minimum sample size to 1000.

For the secondary outcomes of management intentions, reassurance, and acceptability, we compare means and proportions where applicable in the whole sample and generate 95% confidence intervals.

---

[9] Blumenberg, C., Menezes, A.M.B., Gonçalves, H. *et al.* How different online recruitment methods impact on recruitment rates for the web-based *coortesnaweb* project: a randomised trial. *BMC Med Res Methodol* **19**, 127 (2019)

[10] Western Sydney University. Human Research Ethics. Guidance on Reimbursements. 2020.

[11] Dhand, N. K., & Khatkar, M. S. (2014). Statulator: An online statistical calculator. Sample Size Calculator for Comparing Two Independent Means. Accessed 8 May 2023 at htt://statulator.com/SamleSize/ss2M.html

Subgroup analysis
We will compare the effects of the sheets on PrepDM scores in people with chronic pain vs acute pain, and in people with higher pain intensity vs lower pain intensity. We will also examine effects on healthcare intentions in people who received the fact sheet through GP-led approach vs patient-led approach.

Recruitment
Recruitment will happen from receipt of ethics approval (approximately April/May 2023) for a period of up to 12 months or until the target sample size of 1000 is achieved. Participants can opt in via the links on factsheets embedded in HealthShare consultation tools, or via an ad on social media.

GP users of the BetterConsult tool will be initially contacted about the interview/focus group study by email or newsletter from HealthShare. HealthShare regularly communicates with GP users of their tools in this way and have agreed to assist with recruitment. GPs who opt in to be contacted by investigators will receive an invitation email from the research team (Appendix E).

Consent
Participants will access the survey via a QR code on a printed/emailed sheet, via a clickable link on the fact sheet page, or via a link in a social media advertisement. Once they click the link or scan the QR code, participants will be taken to an invitation page summarising the study, with a link to the full PIS which they can download. Those interested in proceeding will complete a digital version of the PCF. Only those clicking 'Yes' to on the digital PCF can access the survey.

GPs who agree to participate in an interview or focus group will receive a link to the participant information sheet and online consent form prior to the interview/focus groups.

Data collection and storage
The survey responses will be collected via The University of Sydney Redcap (for the first two methods: patient-led and GP-led recruitment) and Qualtrics (for social media recruitment). Interviews and focus groups will be conducted and recorded on Zoom and transcribed using Zoom's transcribing feature and will be verified by a researcher. To deidentify Zoom recordings we will only download audio recordings without video. Audio recordings will be assigned a participant number with identifiers kept in a separate database in the University Research Data Store. Once the transcript is verified by the research team, the identifiable recording will be deleted.

Upon completion of the study all data will be stored in The University of Sydney Research Data Store (RDS)

Appendix A – Social media advertisement

People who recently attended primary care for back pain needed for a study conducted by the researchers at the University of Sydney!

We want to hear your views about back pain fact sheets and intentions for future healthcare. The results will help us create better resources for GPs to use with patients who have low back pain. Participation involves a 10-min survey. Click here to find our more!

Appendix B – Fact sheets

COPYRIGHTED MATERIAL REDACTED

Available from: https://www.safetyandquality.gov.au/publications-and-resources/resource-library/how-manage-your-low-back-pain-information-patients

Appendix C

COPYRIGHTED MATERIAL REDACTED

Available from: https://www.safetyandquality.gov.au/publications-and-resources/resource-library/how-manage-your-low-back-pain-information-patients

Appendix D: Clinician interview/focus group guide

Good morning/afternoon/evening. My name is _________________________ from the University of Sydney. I am conducting interviews/focus group to find out about GP views of back pain fact sheets.

Information from these interviews/focus group will help us understand the barriers and facilitators to implementation of fact sheet in primary care.

All of the information we collect in this interview/focus group will be kept confidential. The interview/focus groups will take about 30-60 minutes. I will also be audio-recording the interview/focus group so that I can concentrate on your responses and refer back to them during analysis. Is this okay with you?

If there are any questions you wish to skip or come back to at a later time, or if you need to take a break, please let me know.

Do you have any questions before we begin?

1. Can you tell me a little bit about your practice? [prompts listed below]
   - How many years have you worked as a GP?
   - Have you worked in both private and public sector?
   - What is the average consultation time per patient?

2. Which of the two back pain fact sheets do you prefer and why?
3. Which fact sheets do you think patient find easy to follow and why?
4. Which fact sheets do you think patient find difficult to follow and why?
5. What are the barriers to implementing the fact sheet?
   - Are they easily accessible to GPs?
6. What are the facilitators to implementing the fact sheet?
7. What are the strategies that will increase implementation of these fact sheets?

Thank you for making the time to speak with me.

Thank you again for your time. I wish to remind you that all the information you share with me today will be kept confidential.

Patient interview guide

Good morning/afternoon/evening. My name is _________________________ from the University of Sydney. I am conducting interviews to find out about patient views of back pain fact sheets.

Information from these interviews will help us understand the barriers and facilitators to implementation of fact sheet in primary care.

All of the information we collect in this interview will be kept confidential. The interview should take no longer than 30 minutes. I will also be audio-recording the interview so that I can concentrate on your responses and refer back to them during analysis. Is this okay with you?

If there are any questions you wish to skip or come back to at a later time, or if you need to take a break, please let me know.

Do you have any questions before we begin?

1. Did you read the whole fact sheet?
2. Did you have any problem accessing the fact sheet?

Capability

3. What do you think of the fact sheet? Can you remember how you felt after reading the fact sheet? Did you know much about care for low back pain?
   - Was it easy or hard to follow?
4. Do you feel you know enough about what is recommended for managing back pain now?

Opportunity

5. Did you receive any advice or support from your GP about care for back pain? Did it influence your intention for medical care for back pain?
   - For scan, opioid, using heat, seeking massage/spinal manipulation, staying active

6. Did you feel reassured that there was no serious condition causing your back after reading the fact sheet?
   - What made you feel reassured/not reassured?

Motivation

7. What do you think would be better options to consider first (before scans/opioids) for back pain?

Appendix E: Clinician Recruitment Email/Notice

Newsletter announcement

GPs who treat patients with back pain in primary care needed for a study conducted by researchers at the University of Sydney.
We want to hear your views about back pain fact sheets. Participation involves a 30-60 minute focus group or one-on-one interview. You will be compensated for your time with a gift voucher. If you are interested, please opt in via this link [insert link] or contact the research team directly:

Dr Adrian Traeger: adrian.traeger@sydney.edu.au
Dr Sweekriti Sharma: sweekriti.sharma@sydney.edu.au

Recruitment email from research team for clinician interviews/focus groups for those who opt in

Re: Research project *on back pain fact sheets*: invitation for short interview/focus groups
Attachments:

Dear [insert name of clinician],
Thank you for your interest to participate in the above study from The University of Sydney. The interview/focus groups should take no longer than 30-60 minutes and will be conducted via Zoom. Please find more detailed information about the study and what your participation would involve in the Participant Information Statement.
If you agree to proceed with an interview/focus group, we would appreciate if you could please:

1. Read the Participant Information Statement and click 'Yes' to consent form via this link [*insert REDCap link to Participant Information Statement and Consent Form]*
2. Let us know a suitable date and time when we can schedule an interview/focus group.

We will send you the Zoom meeting invite for the nominated date and time.

Thank you in advance for participating in this project.

We look forward to hearing from you. Please don't hesitate to call Dr Sweekriti Sharma (m: 0481 174 488) or Dr Adrian Traeger (m: 0416122784) any time if you have any questions about the project and your participation.

Kind regards,
Dr Adrian Traeger
Senior Research Fellow
The University of Sydney

Friday, 21 April 2023

Dr Traeger Adrian
School of Public Health: Public Health; Faculty of Medicine and Health
Email: adrian.traeger@sydney.edu.au

Dear Dr Adrian,

The University of Sydney Human Research Ethics Committee (HREC) has considered your application.

I am pleased to inform you that after consideration of your response, your project has been approved.

Details of the approval are as follows:

**Project No.:**	**2023/179**
**Project Title:**	**Back pain fact sheets and intentions for future healthcare**
**Authorised Personnel:**	**Adrian Traeger; Sharma Sweekriti**
**Approval Period:**	**21/04/2023** to **21/04/2027**
**First Annual Report Due:**	**21/04/2024**

Documents Approved:

Date Uploaded	Version Number	Document Name
06/04/2023	Version 2	Protocol_Clean
06/04/2023	Version 2	Consent form_Clean
06/04/2023	Version 2	Participant Info Statement_Clean
24/02/2023	Version 1	Questionnaire

Condition/s of Approval

- Research must be conducted according to the approved proposal.
- An annual progress report must be submitted to the Ethics Office on or before the anniversary of approval and on completion of the project.
- You must report as soon as practicable anything that might warrant review of ethical approval of the project including:
  - Serious or unexpected adverse events (which should be reported within 72 hours).
  - Unforeseen events that might affect continued ethical acceptability of the project.
- Any changes to the proposal must be approved prior to their implementation (except where an amendment is undertaken to eliminate *immediate* risk to participants).
- Personnel working on this project must be sufficiently qualified by education, training and experience for their role, or adequately supervised. Changes to personnel must be reported and approved.
- Personnel must disclose any actual or potential conflicts of interest, including any financial or other interest or affiliation, as relevant to this project.
- Data and primary materials must be retained and stored in accordance with the relevant legislation and University guidelines.
- Ethics approval is dependent upon ongoing compliance of the research with the *National Statement on Ethical Conduct in Human Research*, the *Australian Code for the Responsible Conduct of*

Research, applicable legal requirements, and with University policies, procedures and governance requirements.

- The Ethics Office may conduct audits on approved projects.
- The Chief Investigator has ultimate responsibility for the conduct of the research and is responsible for ensuring all others involved will conduct the research in accordance with the above.

This letter constitutes ethical approval only.

Please contact the Ethics Office should you require further information or clarification.

Sincerely,

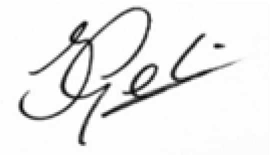

Associate Professor Ingrid Gelissen
Acting Chair, Health Review Committee (Low Risk)

The University of Sydney of Sydney HRECs are constituted and operate in accordance with the National Health and Medical Research Council's (NHMRC) National Statement on Ethical Conduct in Human Research (2018) and the NHMRC's Australian Code for the Responsible Conduct of Research (2018)

Monday, 22 May 2023

Dr Adrian C Traeger
School of Public Health: Public Health; Faculty of Medicine and Health
Email: adrian.traeger@sydney.edu.au

Dear Adrian C,

Your request to modify this project, which was submitted on 9/5/2023, has been considered.

This project has been approved to proceed with the proposed amendments.

Protocol Number: **2023/179**
Protocol Title: **Back pain fact sheets and decisions about future healthcare**

Annual Report Due: **9/5/2023**

Documents Approved:

Date Uploaded	Version Number	Document Name
09/05/2023	Version 3	Cover letter
09/05/2023	Version 2	Questionnaire_Clean
09/05/2023	Version 3	Study Protocol_Clean

Please contact the ethics office should you require further information.

Sincerely,

Dr Marinda Taha
Chair
Modification Review Committee Chair (MRC 1)

The University of Sydney of Sydney HRECs are constituted and operate in accordance with the National Health and Medical Research Council's (NHMRC) National Statement on Ethical Conduct in Human Research (2018) and the NHMRC's Australian Code for the Responsible Conduct of Research (2018)

Friday, 10 November 2023

Dr Adrian C Traeger
School of Public Health: Public Health; Faculty of Medicine and Health
Email: adrian.traeger@sydney.edu.au

Dear Adrian C,

Your request to modify this project, which was submitted on 17/10/2023, has been considered.

This project has been approved to proceed with the proposed amendments.

Protocol Number: **2023/179**
Protocol Title: **Back pain fact sheets and decisions about future healthcare**

Documents Approved:

Date Uploaded	Version Number	Document Name
17/10/2023	Version 7	HealthShare JAMA sheet Protocol_V7_17.10.2023_Tracked

Please contact the ethics office should you require further information.

Sincerely,

Dr Marinda Taha
Chair
Modification Review Committee Chair (MRC 1)

The University of Sydney of Sydney HRECs are constituted and operate in accordance with the National Health and Medical Research Council's (NHMRC) National Statement on Ethical Conduct in Human Research (2018) and the NHMRC's Australian Code for the Responsible Conduct of Research (2018)

TITLE

Back pain fact sheets and intentions for future healthcare

Investigators

Adrian C Traeger (Senior Research Fellow, The University of Sydney)

Swee Sharma (Research Fellow, The University of Sydney)

BACKGROUND

Patient education improves outcomes for people with low back pain. There is high quality evidence that written or verbal patient education can reassure patients and reduce subsequent healthcare utilisation in the short- and long-term.[1] It is the most common recommendation in international clinical guidelines for back pain. Yet primary care clinicians globally appear to underuse patient education.[2]

Easily accessed, evidence-based fact sheets, that can be printed or emailed to patients, could be a simple option to improve care for low back pain.[3] A Cochrane review found patient-mediated interventions, including access to written patient materials, may improve the appropriateness of care for mixed conditions, though the evidence was uncertain.[4]

Digital health companies now provide consultation software add-ons that provide access to fact sheets during a consultation. SMS systems allow clinicians to routinely send fact sheets following a consultation. Two new fact sheets being implemented using these approaches in Australia in 2023 are the *JAMA Patient Page* (JAMA fact sheet) and the *Australian Commission for Healthcare Safety and Quality Information for Patients Fact Sheet* (ACHSQ fact sheet). Both resources are evidence-based but differ in their focus. A key difference is that the JAMA fact sheet focuses on listing evidence-based treatment options, whereas the ACHSQ fact sheet explains how a person can self-manage back pain.

There is no rigorous evidence on the effects of either of these new resources among people seeking care for low back pain. It is uncertain whether a fact sheet resource that focuses more on listing medical management options (JAMA fact sheet), can increase a patient's intention to use medical care, compared with a resource that focuses on self-management.

AIMS

Our primary aim is to compare testing and treatment intentions among people seeking care for low back pain who are given a fact sheet. Secondary aims are to examine feelings of reassurance about serious pathology and the acceptability of the fact sheets to people seeking care for low back pain.

METHODS

Consumer involvement

---

[1] Traeger AC, Hübscher M, Henschke N, et al. JAMA Intern Med. 2015 May;175(5):733-43

[2] Traeger AC, et al. Bull World Health Organ. 2019:97(6):423-433.

[3] Hébert ET, Caughy MO, Shuval K. Br J Sports Med. 2012 Jul;46(9):625-31.

[4] Fønhus et al. Cochrane Database Syst Rev. 2018 Sep 11;9(9):CD012472.

Our consumer partner CI McBride explained to the research team the value of receiving high-quality information about low back pain from one's GP. CI McBride helped us design our patient survey and suggested we document whether patients recalled their GP explaining the content of the fact sheet.

Design, participants, and setting

This will be a survey of people receiving one of the two fact sheets in primary care. Around 400 patients presenting with back pain to GP practices across Australia will be surveyed. Participants could receive a fact sheet via two methods. The first method is directly from their GP who prints and/or emails the sheet to their patient (GP-led access, Figure 1). The GP determines which sheet they will provide and so receipt of the JAMA or ACHSQ fact sheet is non-random.

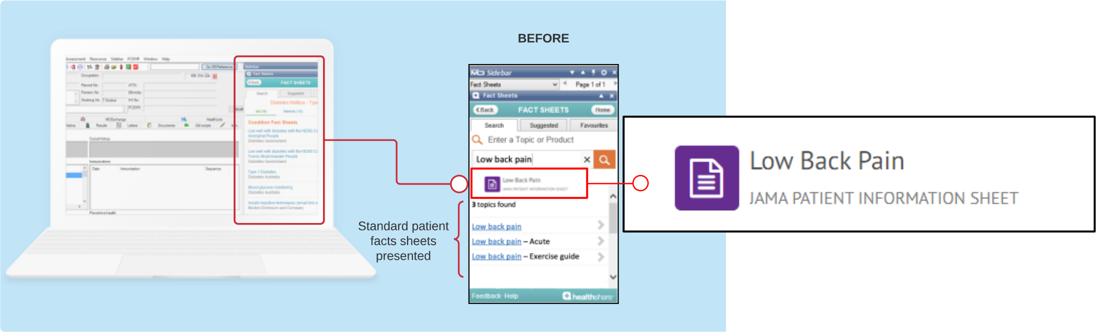

Figure 1. GP-led access to back pain fact sheet. Appearance of the HealthShare sidebar where GPs can access fact sheets that are relevant to what is typed into consultation software. A clinician can print or email the sheet to their patient as they wish.

The second method is patient-led access to a back pain fact sheet via an SMS system called BetterConsult. BetterConsult invites a patient to receive a back pain fact sheet after they see their GP (Figure 2). Whether the patient receives the JAMA or ACHSQ fact sheet is determined randomly by the fact sheet distributor, HealthShare. Patients can view the sheet on their smartphone or print the sheet out and their GP will be notified. GPs can open and discuss the fact sheet with their patient during the consult if they wish. Twenty-four hours after their consultation with the GP, HealthShare sends patients an SMS containing a link to the fact sheet.

The nature of these two access methods means that we can make a randomised comparison of the survey responses to the two fact sheets delivered via patient-led access, but not those delivered by the GP-led access, ie in the consult by print or email. Data from patients receiving a fact sheet using these two methods (GP-led access vs Patient-led access) will therefore be analysed separately.

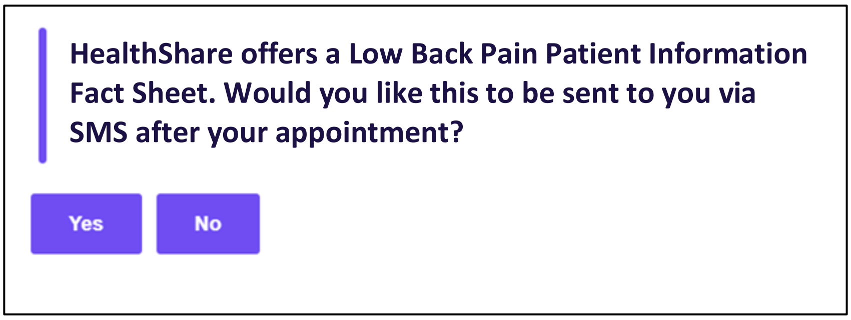

Figure 2. Patient-led access to back pain fact sheet. Patient opts to receive a fact sheet via SMS when they complete their details in a pre-consultation tool (BetterConsult).

Procedure

Participants will be people >18 years visiting their GP for back pain who receive a back pain fact sheet and who choose to complete the online survey. Patients who access the survey link and agree to participate in the study will provide clinical and demographic details via an online form (age, sex, ethnicity, duration of back pain, pain intensity). No data from a participant’s medical record will be used in this study.

COPYRIGHTED MATERIAL REDACTED

Figure 3. Potential participants can access the survey via a QR code on printed/emailed sheet or via clickable link on the fact sheet page.

Outcomes

The **primary outcome** of this study is future intentions regarding non-recommended medical care for low back pain, adapted from Hersch et al.[5] and collected immediately after the participant reads the information:

- At the moment, which of the following best describes your intentions about **having medical imaging (x-ray, CT, MRI)** for your low back pain?
  Response options:
  - I definitely will have medical imaging
  - I am likely to have medical imaging
  - I am unsure
  - I am NOT likely to have medical imaging
  - I definitely will NOT have medical imaging
- At the moment, which of the following best describes your intentions about **taking opioids [prescription medicines e.g. oxycodone]** for your low back pain?
  - I definitely will take opioids
  - I am likely to take opioids
  - I am unsure
  - I am NOT likely to take opioids
  - I definitely will NOT take opioids
- At the moment, which of the following best describes your intentions about **using non-drug approaches (physical activity/exercise, heat packs, massage)** for your low back pain?
  - I definitely will use non-drug approaches
  - I am likely to use non-drug approaches
  - I am unsure
  - I am NOT likely to use non-drug approaches
  - I definitely will NOT use non-drug approaches

Participants who report being definitely/likely to seek imaging or opioids for their low back pain will be classified as having "intentions for medical care". We will then estimate the proportion of people with intentions for medical care in those who read the JAMA fact sheet vs those who read the ACHSQ fact sheet.

Secondary outcomes

- Reassurance
  - How reassured are you that there is no serious condition causing your low back pain? (from Traeger et al)[6] 1 - not reassured at all; 10 - completely reassured

- Acceptability
  - Did you read the fact sheet all the way through?
    If Yes - About how long did you spend reading the fact sheet?

[5] Hersch J, et al. BMJ Open. 2014 May 15;4(5):e004990.
[6] Traeger AC et al. JAMA Neurol. 2019 Feb 1;76(2):161-169.

[_______ min] .

If No - How much of the fact sheet did you read?

[options: most / some / a little]

- How would you rate the length of the fact sheet?
  [options: much too short / a little too short / just about right / a little too long / much too long]

- How much of the information in the fact sheet was new to you?
  [options: all / most / some / none]

- How balanced did you find the fact sheet? Did you feel it was..?
  [options: clearly slanted towards medical care / a little slanted towards medical care / completely balanced / a little slanted away from medical care / clearly slanted away from medical care]

- How strongly do you agree or disagree with each of the following statements.
  [options: strongly agree / agree / neither agree nor disagree / disagree / strongly disagree]

  - I found the information in the fact sheet clear and easy to understand.
  - I found the fact sheet helpful in making my decision about medical care for back pain.
  - I would recommend this fact sheet to other people who are considering their options to treat low back pain.

- Fidelity
  - Which of the following sheets did you receive? [participant selects image of JAMA or ACSHQ]
  - How did you receive the sheet
    - From my GP
    - Via SMS from my GP practice
    - Other [please describe]

Exposures

Exposures will be two recently published, evidence-based resources for low back pain. The *JAMA Patient Page* (Available from: https://jamanetwork.com/journals/jama/fullarticle/2782184) was published on 20/7/21 and describes guideline-endorsed medical and non-medical management options for low back pain.

The *Australian Commission for Healthcare Safety and Quality (ACHSQ) Information for Patients Fact Sheet* (Available from: https://www.safetyandquality.gov.au/publications-and-resources/resource-library/how-manage-your-low-back-pain-information-patients) was published on 22/9/22 and describes how to self-manage low back pain.

Both factsheets have been implemented into routine practice via HealthShare consultation tools.

Statistical analysis
Our primary analysis will compare the number and proportion of people with intentions for medical care in people exposed to the JAMA fact sheet vs the ACHSQ fact sheet. Assuming a baseline proportion of 50% with intentions for medical care,[7] to detect a difference of 10% (ie 50% in JAMA fact sheet group, 40% in ACHSQ group) with 80% power, we require a sample size of 385. To account for anticipated uneven group size we will increase the minimum sample size to 500.

For the secondary outcomes of reassurance and acceptability, we compare means and proportions where applicable in the whole sample, and generate 95% confidence intervals.

Subgroup analysis
We will conduct a planned subgroup analysis to examine healthcare intentions in people who received the fact sheet through GP-led approach vs patient-led approach.

Recruitment
Recruitment will happen from receipt of ethics approval (approximately April/May 2023) for a period of up to 12 months or until the target sample size of 500 is achieved.

Consent
Participants will access the survey via a QR code on a printed/emailed sheet or via a clickable link on the fact sheet page. Once they click the link or scan the QR code, participants will be taken to an invitation page summarising the study, with a link to the full PIS which they can download. Those interested in proceeding will complete a digital version of the PCF. Only those clicking 'Yes' to on the digital PCF can access the survey.

Data storage
Upon completion of the study all data will be stored in The University of Sydney Research Data Store (RDS)

Appendix A

[7] Jenkins HJ et al Eur J Pain. 2016 Apr;20(4):573-80.

COPYRIGHTED MATERIAL REDACTED

Available from: https://jamanetwork.com/journals/jama/fullarticle/2782184

COPYRIGHTED MATERIAL REDACTED

Available from: https://www.safetyandquality.gov.au/publications-and-resources/resource-library/how-manage-your-low-back-pain-information-patients

TITLE

Back pain fact sheets and decisions about future healthcare

Investigators

Adrian C Traeger (Senior Research Fellow, The University of Sydney)
Swee Sharma (Research Fellow, The University of Sydney)

BACKGROUND

Patient education improves outcomes for people with low back pain. There is high quality evidence that written or verbal patient education can reassure patients and reduce subsequent healthcare utilisation in the short- and long-term.[1] It is the most common recommendation in international clinical guidelines for back pain. Yet primary care clinicians globally appear to underuse patient education.[2]

Easily accessed, evidence-based fact sheets, that can be printed or emailed to patients, could be a simple option to improve care for low back pain.[3] A Cochrane review found patient-mediated interventions, including access to written patient materials, may improve the appropriateness of care for mixed conditions, though the evidence was uncertain.[4] Fact sheets that help patients prepare for decision-making about their treatment options could encourage shared decision-making and better care.[5]

Digital health companies now provide consultation software add-ons that provide access to fact sheets during a consultation. SMS systems allow clinicians to routinely send fact sheets following a consultation. Two new fact sheets being implemented using these approaches in Australia in 2023 are the *JAMA Patient Page* (JAMA fact sheet) and the *Australian Commission for Healthcare Safety and Quality Information for Patients Fact Sheet* (ACHSQ fact sheet). Both resources are evidence-based but differ in their focus. A key difference is that the JAMA fact sheet focuses on listing evidence-based treatment options, whereas the ACHSQ fact sheet explains how a person can self-manage back pain.

There is no rigorous evidence on the effects of either of these new resources among people seeking care for low back pain. It is uncertain whether a fact sheet resource that focuses more on listing medical management options (JAMA fact sheet), can increase a patient's preparedness for decision-making, compared with a resource that focuses on self-management.

AIMS

Our primary aim is to compare preparation for decision-making among people seeking care for low back pain who are given one of two fact sheets. Secondary aims are to examine

[1] Traeger AC, Hübscher M, Henschke N, et al. JAMA Intern Med. 2015 May;175(5):733-43
[2] Traeger AC, et al. Bull World Health Organ. 2019:97(6):423-433.
[3] Hébert ET, Caughy MO, Shuval K. Br J Sports Med. 2012 Jul;46(9):625-31.
[4] Fønhus et al. Cochrane Database Syst Rev. 2018 Sep 11;9(9):CD012472.
[5] Bennett C, Graham ID, Kristjansson E, et al. Patient Educ Couns. 2010 Jan;78(1):130-3.

managment intentions, feelings of reassurance about serious pathology and the acceptability of the fact sheets to people seeking care for low back pain.

METHODS

Consumer involvement

Our consumer partner CI McBride explained to the research team the value of receiving high-quality information about low back pain from one's GP. CI McBride helped us design our patient survey and suggested we document whether patients recalled their GP explaining the content of the fact sheet.

Design, participants, and setting

This will be a survey of people receiving one of the two fact sheets in primary care. Around 790 patients presenting with back pain to GP practices across Australia will be surveyed. Participants could receive a fact sheet via two methods. The first method is directly from their GP who prints and/or emails the sheet to their patient (GP-led access, Figure 1). The GP determines which sheet they will provide and so receipt of the JAMA or ACHSQ fact sheet is non-random.

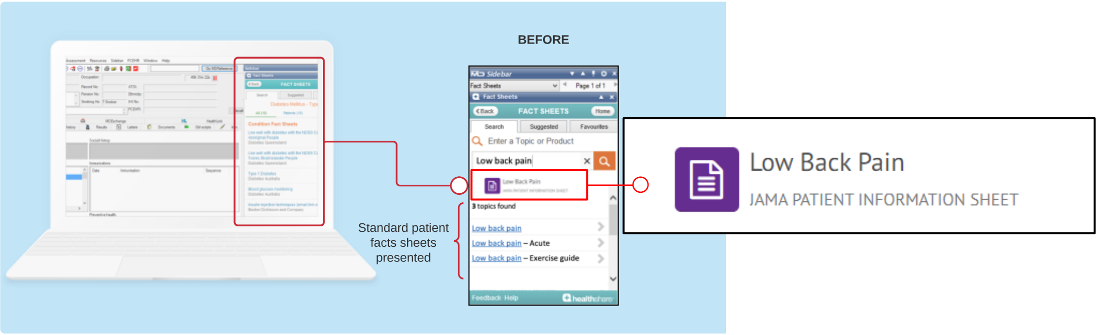

Figure 1. GP-led access to back pain fact sheet. Appearance of the HealthShare sidebar where GPs can access fact sheets that are relevant to what is typed into consultation software. A clinician can print or email the sheet to their patient as they wish.

The second method is patient-led access to a back pain fact sheet via an SMS system called BetterConsult. BetterConsult invites a patient to receive a back pain fact sheet after they see their GP (Figure 2). Whether the patient receives the JAMA or ACHSQ fact sheet is determined randomly by the fact sheet distributor, HealthShare. Patients can view the sheet on their smartphone or print the sheet out and their GP will be notified. GPs can open and discuss the fact sheet with their patient during the consult if they wish. Twenty-four hours after their consultation with the GP, HealthShare sends patients an SMS containing a link to the fact sheet.

The nature of these two access methods means that we can make a randomised comparison of the survey responses to the two fact sheets delivered via patient-led access, but not those delivered by the GP-led access, ie in the consult by print or email. Data from patients receiving a fact sheet using these two methods (GP-led access vs Patient-led access) will therefore be analysed separately.

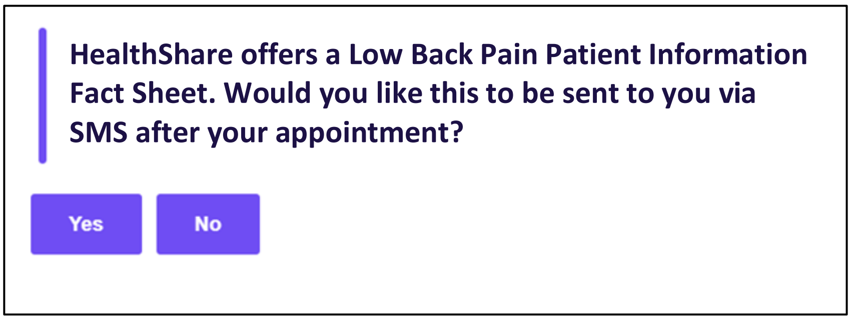

Figure 2. Patient-led access to back pain fact sheet. Patient opts to receive a fact sheet via SMS when they complete their details in a pre-consultation tool (BetterConsult).

Procedure

Participants will be people >18 years visiting their GP for back pain who receive a back pain fact sheet and who choose to complete the online survey. Patients who access the survey link and agree to participate in the study will provide clinical and demographic details via an online form (age, sex, ethnicity, duration of back pain, pain intensity). No data from a participant's medical record will be used in this study.

COPYRIGHTED MATERIAL REDACTED

Figure 3. Potential participants can access the survey via a QR code on printed/emailed sheet or via clickable link on the fact sheet page.

Outcomes

The **primary outcome** of this study is preparation for decision-making, measured using the Preparation for Decision Making (PrepDM) scale.[6] PrepDM has 10-items that address concepts of preparedness for decision-making. It has successfully discriminated between different educational resources in randomised trials and is valid and reliable.

Secondary outcomes

- Management intentions
  We adapted items from Hersch et al.[7] for decisions about care for low back pain:
  - At the moment, which of the following best describes your intentions about **having a scan (x-ray, CT, MRI)** for your low back pain?
    Response options:
    - I definitely will have a scan
    - I am likely to have a scan
    - I am unsure
    - I am NOT likely to have a scan
    - I definitely will NOT have a scan

    Could you please tell us more about why you selected [the option above]?
    [free text box]

  - At the moment, which of the following best describes your intentions about **taking opioids (prescription pain medicines e.g. oxycodone, codeine, tramadol)** for your low back pain?
    - I definitely will take opioids
    - I am likely to take opioids
    - I am unsure
    - I am NOT likely to take opioids
    - I definitely will NOT take opioids

  - Could you please tell us more about why you selected [the option above]?
    [free text box]

  - At the moment, which of the following best describes your intentions about **using heat (e.g. heat packs, hot water bottle)** for your low back pain?
    - I definitely will use heat
    - I am likely to use heat

[6] Bennett C, Graham ID, Kristjansson E, et al. Patient Educ Couns. 2010 Jan;78(1):130-3
[7] Hersch J, et al. BMJ Open. 2014 May 15;4(5):e004990.

    - I am unsure
    - I am NOT likely to use heat
    - I definitely will NOT use heat

  - At the moment, which of the following best describes your intentions about **seeking massage and/or spinal manipulation (e.g. from a physiotherapist or chiropractor)** for your low back pain?
    - I definitely will seek massage and/or spinal manipulation
    - I am likely to seek massage and/or spinal manipulation
    - I am unsure
    - I am NOT likely to seek massage and/or spinal manipulation
    - I definitely will NOT seek massage and/or spinal manipulation

  - At the moment, which of the following best describes your intentions about **staying active** while you have low back pain?
    - I definitely will stay active
    - I am likely to stay active
    - I am unsure
    - I am NOT likely to stay active
    - I definitely will NOT stay active

Participants who report being definitely/likely to seek imaging or opioids for their low back pain will be classified as having "intentions for medical care".

- Reassurance
  - How reassured are you that there is no serious condition causing your low back pain? (from Traeger et al)[8] 0 - not reassured at all; 10 - completely reassured

- Acceptability
  - Did you read the fact sheet all the way through?

    If Yes - how many minutes did you spend reading the fact sheet?

    [_______ min] .

    If No - How much of the fact sheet did you read?

    [options: most / some / a little]

  - How would you rate the length of the fact sheet?

    [options: much too short / a little too short / just about right / a little too long / much too long]

  - How much of the information in the fact sheet was new to you?

    [options: all / most / some / none]

[8] Traeger AC et al. JAMA Neurol. 2019 Feb 1;76(2):161-169.

- How balanced did you find the fact sheet? For example, with regard to emphasis on the need for **medical care** (pain medicines, imaging, GP appointments, specialist care, surgery), did you feel it was..?
  [options: very biased towards medical care / somewhat biased towards medical care / balanced / somewhat biased against medical care / very biased against medical care]

- How strongly do you agree or disagree with each of the following statements.
  [options: strongly agree / agree / neither agree nor disagree / disagree / strongly disagree]

  - I found the information in the fact sheet clear and easy to understand.
  - I found the fact sheet helpful in making my decision about medical care for back pain.
  - I would recommend this fact sheet to other people who are considering their options to treat low back pain.

- Fidelity
  - How did you receive the fact sheet
    - From my GP
    - Via SMS from my GP practice
    - Other [please describe]

Exposures
Exposures will be two recently published, evidence-based resources for low back pain. The *JAMA Patient Page* (Available from: https://jamanetwork.com/journals/jama/fullarticle/2782184) was published on 20/7/21 and describes guideline-endorsed medical and non-medical management options for low back pain.

The *Australian Commission for Healthcare Safety and Quality (ACHSQ) Information for Patients Fact Sheet* (Available from: https://www.safetyandquality.gov.au/publications-and-resources/resource-library/how-manage-your-low-back-pain-information-patients) was published on 22/9/22 and describes how to self-manage low back pain.
Both factsheets have been implemented into routine practice via HealthShare consultation tools.

Statistical analysis
Our primary analysis will compare the PrepDM scores in people exposed to the JAMA fact sheet vs the ACHSQ fact sheet, immediately after they have read the sheet. Assuming a mean difference of 6 points on a 100-point scale (ie 60 points in JAMA fact sheet group, 54

points in ACHSQ group, SD of 26) with 90% power, we require a sample size of 790.[9] To account for anticipated uneven group size we will increase the minimum sample size to 1000.

For the secondary outcomes of management intentions, reassurance, and acceptability, we compare means and proportions where applicable in the whole sample and generate 95% confidence intervals.

Subgroup analysis

We will conduct a planned subgroup analysis to examine healthcare intentions in people who received the fact sheet through GP-led approach vs patient-led approach, people with chronic pain vs acute pain, and in people with high pain intensity (≥6/10) vs lower pain intensity.

Recruitment

Recruitment will happen from receipt of ethics approval (approximately April/May 2023) for a period of up to 12 months or until the target sample size of 1000 is achieved.

Consent

Participants will access the survey via a QR code on a printed/emailed sheet or via a clickable link on the fact sheet page. Once they click the link or scan the QR code, participants will be taken to an invitation page summarising the study, with a link to the full PIS which they can download. Those interested in proceeding will complete a digital version of the PCF. Only those clicking 'Yes' to on the digital PCF can access the survey.

Data storage

Upon completion of the study all data will be stored in The University of Sydney Research Data Store (RDS)

[9] Dhand, N. K., & Khatkar, M. S. (2014). Statulator: An online statistical calculator. Sample Size Calculator for Comparing Two Independent Means. Accessed 8 May 2023 at htt://statulator.com/SamleSize/ss2M.html

Appendix A

COPYRIGHTED MATERIAL REDACTED

Available from: https://jamanetwork.com/journals/jama/fullarticle/2782184

Appendix B

COPYRIGHTED MATERIAL REDACTED

Available from: https://www.safetyandquality.gov.au/publications-and-resources/resource-library/how-manage-your-low-back-pain-information-patients

ANZCTR
Australian New Zealand Clinical Trials Registry

LOG OFF
Logged in as Sweekriti Sharma
[Account details]

DEFINITIONS HINTS AND TIPS FAQs REGISTER TRIAL MY TRIALS

Register a trial

To achieve prospective registration, we recommend submitting your trial for registration at the same time as ethics submission.

Updating a registered trial?

New videos with **general tips for updating**, and how to **update recruitment status (Step 7)** are now available!

Acknowledgment | Step 1: Titles & IDs | Step 2: Health condition | Step 3: Intervention/exposure
Step 4: Outcomes | Step 5: Eligibility | Step 6: Study design | Step 7: Recruitment
Step 8: Funding & Sponsors | Step 9: Ethics & Summary | Step 10: Contacts | Step 11: Data sharing statement
Step 12: Summary Results | Review & Submit

You must complete all mandatory fields on step(s) 12 before you are able to submit this trial.

Request number	385954
Current page	Review

< BACK SUBMIT

**Registration number**	ACTRN12623000603617
**Ethics application status**	Approved
**Date submitted**	24/05/2023
**Date registered**	2/06/2023
**Date data sharing statement initially provided**	2/06/2023
**Type of registration**	Prospectively registered

Titles & IDs

Edit step 1

**Public title**	Back pain fact sheets and decisions about future healthcare
**Scientific title**	Effect of back pain fact sheets on decisions about future healthcare in adults attending primary care
**Secondary ID [1]**	None
**Universal Trial Number (UTN)**	
**Trial acronym**	
**Linked study record**	

Health condition

Edit step 2

Health condition(s) or problem(s) studied:

low back pain

Condition category	Condition code
Musculoskeletal	Other muscular and skeletal disorders
Public Health	Health promotion/education

Intervention/exposure

Edit step 3

Study type

Interventional

Description of intervention(s) / exposure

JAMA Patient Page on low back pain. 1 page fact sheet delivered by GP or by SMS from GP practice. Content focuses on listing evidence-based treatment options. Freely available resource accessible at: https://jamanetwork.com/journals/jama/fullarticle/2782184

Participants can read the fact sheet at their own time and pace. GPs can open and discuss the fact sheet with their patient during the consult if they wish. Twenty-four hours after their consultation with the GP, HealthShare sends patients an SMS containing a link to the fact sheet on behalf of the GP. To monitor adherence participants will complete a study-specific questionnaire.

Update

JAMA Patient Page on low back pain. 1 page fact sheet delivered by GP or by SMS from GP practice. Content focuses on listing evidence-based treatment options. Freely available resource accessible at: https://jamanetwork.com/journals/jama/fullarticle/2782184

Participants can read the fact sheet at their own time and pace. GPs can open and discuss the fact sheet with their patient during the consult if they wish. Twenty-four hours after their consultation with the GP, HealthShare sends patients an SMS containing a link to the fact sheet on behalf of the GP. To monitor adherence participants will complete a study-specific questionnaire.

Participants can also access the fact sheet via an advertisement on social media (Facebook, Twitter, and Instagram). The advertisement will invite people who have recently seen their GP for back pain to view a fact sheet and complete a short survey.

Exposure: We are adding nested study within the original study to understand recruitment rates using ABACAD replicated time series design. Control Period A – Advertisement is a clickable link and QR code with the words "Have your say about this fact sheet" in a red box in the top right corner of the fact sheet. Intervention Period B – Advertisement is a clickable link that mentions a financial incentive to participate: "Have your say about this fact sheet. Complete a short survey for your chance to win" is written in a red box in the top right corner of the fact sheet. Intervention Period C – Advertisement is a clickable link with phrasing that appeals to social good: "Help us improve fact sheets for people with back pain." Intervention Period D – Advertisement is a clickable link with phrasing that appeals to both social good and provides financial incentive: "Help us improve fact sheets for people with back pain. Complete a short survey for your chance to win" in a red box in the top right corner of the fact sheet.

Reason

We added a another method of recruiting participants via social media advertisement because with the previous methods we are not getting enough participants. We also added a nested study with the original study to understand recruitment rates.

Intervention code [1]

Treatment: Other

Comparator / control treatment

Australian Commission for Healthcare Safety and Quality Information for Patients Fact Sheet (ACHSQ fact sheet), 1 page fact sheet delivered by GP or by SMS from GP practice. Content focuses on explaining how a person can self-manage back pain. Freely available resource accessible at: https://www.safetyandquality.gov.au/publications-and resources/resource-library/how-manage-your-low-back-pain-information-patients

Participants can read the fact sheet at their own time and pace. GPs can open and discuss the fact sheet with their patient during the consult if they wish. Twenty-four hours after their consultation with the GP, HealthShare sends patients an SMS containing a link to the fact sheet on behalf of the GP. To monitor adherence participants will complete a study-specific questionnaire.

Update

Australian Commission for Healthcare Safety and Quality Information for Patients Fact Sheet (ACHSQ fact sheet), 1 page fact sheet delivered by GP or by SMS from GP practice. Content focuses on explaining how a person can self-manage back pain. Freely available resource accessible at: https://www.safetyandquality.gov.au/publications-and resources/resource-library/how-manage-your-low-back-pain-information-patients

Participants can read the fact sheet at their own time and pace. GPs can open and discuss the fact sheet with their patient during the consult if they wish. Twenty-four hours after their consultation with the GP, HealthShare sends patients an SMS containing a link to the fact sheet on behalf of the GP. To monitor adherence participants will complete a study-specific questionnaire.

	Participants can also access the fact sheet via an advertisement on social media (Facebook, Twitter, and Instagram). The advertisement will invite people who have recently seen their GP for back pain to view a fact sheet and complete a short survey.
Reason	We added a another method of recruiting participants via social media advertisement because with the previous methods we are not getting enough participants.
**Control group**	Active

Outcomes

Edit step 4

**Primary outcome [1]**	Preparation for decision-making, measured using the Preparation for Decision Making (PrepDM) scale.
Timepoint [1]	Immediate (after reading fact sheet)
**Secondary outcome [1]**	Management intentions assessed by study-specific questionnaire
Timepoint [1]	Immediate (after reading fact sheet)
**Secondary outcome [2]**	Reassurance assessed by study-specific questionnaire
Timepoint [2]	Immediate (after reading fact sheet)
**Secondary outcome [3]**	Acceptability assessed by study-specific questionnaire
Timepoint [3]	Immediate (after reading fact sheet)
**Secondary outcome [4]**	Fidelity assessed by study-specific questionnaire
Timepoint [4]	Immediate (after reading fact sheet)
**Secondary outcome [5]**	[New secondary outcome]
Update	Implementation via semi-structured interviews and or focus groups (depending on feasibility) with up to 20 clinicians and 20 patients (interview only) to identify barriers and facilitators to implementation of the fact sheets in primary care.
Reason	To identify barriers and facilitators to implementation of the fact sheets in primary care.
Timepoint [5]	[New secondary outcome]
Update	For patient interview: immediately after patient participants complete the survey. For clinicians interview or focus group: after the intervention period
Reason	To identify barriers and facilitators to implementation of the fact sheets in primary care.
**Secondary outcome [6]**	[New secondary outcome]
Update	Recruitment rates by measuring proportion of people exposed to the survey advertisement who consent to participate.
Reason	To understand recruitment rates of social media advertisement
Timepoint [6]	[New secondary outcome]
Update	After the recruitment is complete
Reason	To understand recruitment rates of social media advertisement

Eligibility

Edit step 5

**Key inclusion criteria**	1. People 18 years or older<br>2. Visiting their GP for back pain<br>3. Receive a back pain fact sheet and choose to complete the online survey
**Minimum age**	18 Years
**Maximum age**	No limit
**Sex**	Both males and females
**Can healthy volunteers participate?**	No
**Key exclusion criteria**	Nil

Study design

Edit step 6

**Purpose of the study**	Educational / counselling / training

Allocation to intervention
Randomised controlled trial

Procedure for enrolling a subject and allocating the treatment (allocation concealment procedures)
Allocation is not concealed

Methods used to generate the sequence in which subjects will be randomised (sequence generation)
This study is quasi randomised. Participants are allocated to intervention and control groups via two methods. The first method is based on GP discretion as to which study fact sheet they provide to their patient. The comparison of outcomes for people allocated to fact sheet interventions via this method is therefore non-random. The second allocation method is random. Based on a computerised random number list, a GP software provider will allocate access to either the intervention or control fact sheet, for one month at a time for the duration of the trial. The comparison of outcomes for people allocated to interventions via this method is therefore based on a randomised computer generated sequence.

Update
This study is quasi randomised. Participants are allocated to intervention and control groups via three methods. The first method is based on GP discretion as to which study fact sheet they provide to their patient. The comparison of outcomes for people allocated to fact sheet interventions via this method is therefore non-random. The second allocation method is random. Based on a computerised random number list, a GP software provider will allocate access to either the intervention or control fact sheet, for one month at a time for the duration of the trial. The comparison of outcomes for people allocated to interventions via this method is therefore based on a randomised computer generated sequence.
The third allocation method will be random. Participants via social media advertisement will be allocated to either intervention or control fact sheet randomly.

Reason
We added a third method of recruitment via social media advertisement because we were not getting enough participants.

Masking / blinding
Open (masking not used)

Who is / are masked / blinded?

Intervention assignment
Parallel

Other design features

Phase
Not Applicable

Type of endpoint/s
Efficacy

Statistical methods / analysis
Our primary analysis will compare the PrepDM scores in people exposed to the JAMA fact sheet vs the ACHSQ fact sheet, immediately after they have read the sheet. Assuming a mean difference of 6 points on a 100-point scale (ie 60 points in JAMA fact sheet group, 54 points in ACHSQ group, SD of 26) with 90% power, we require a sample size of 790. To account for anticipated uneven group size we will increase the minimum sample size to 1000.

For the secondary outcomes of management intentions, reassurance, and acceptability, we compare means and proportions where applicable in the whole sample and generate 95% confidence intervals.

Subgroup analysis
We will compare the effects of the sheets on PrepDM scores in people with chronic pain vs acute pain, and in people with higher pain intensity vs lower pain intensity. We will also examine effects on healthcare intentions in people who received the fact sheet through GP-led approach vs patient-led approach.

Data from patients receiving a fact sheet using random and non-random methods of allocation will be analysed separately in a sensitivity analysis.

Update
Our primary analysis will compare the PrepDM scores in people exposed to the JAMA fact sheet vs the ACHSQ fact sheet, immediately after they have read the sheet. Assuming a mean difference of 6 points on a 100-point scale (ie 60 points in JAMA fact sheet group, 54 points in ACHSQ group, SD of 26) with 90% power, we require a sample size of 790. To account for anticipated uneven group size we will increase the minimum sample size to 1000.

For the secondary outcomes of management intentions, reassurance, and acceptability, we compare means and proportions where applicable in the whole sample and generate 95% confidence intervals.

Subgroup analysis
We will compare the effects of the sheets on PrepDM scores in people with chronic pain vs acute pain, and in people with higher pain intensity vs lower pain intensity. We will also examine effects on healthcare intentions in people who received the fact sheet through GP-led approach vs patient-led approach.

Data from patients receiving a fact sheet using random and non-random methods of allocation will be analysed separately in a sensitivity analysis.

Descriptive analyses will be done to report the response rate for all participants according to trial period.

We will compare the number and proportion of participants responding to messages.

Reason We want to understand different survey advertising methods using fact sheets embedded in consulation software (BetterConsult provided by the company HealthShare) on recruitment rate.

Recruitment

Edit step 7

Recruitment status Not yet recruiting

Update Recruiting

Reason We added a new method of recruitment. Recruitment via a new method hasn't started. Recruitment via preiously approved method has started.

Date of first participant enrolment

Anticipated 9/06/2023

Update 2/06/2023

Reason We added a new method of recruitment via social media advertisement. The ethics amendment has been approved now.

Actual

Update 2/06/2023

Reason We added a new method of recruitment via social media advertisement. The ethics amendment has been approved now.

Date of last participant enrolment

Anticipated 7/06/2024

Actual

Date of last data collection

Anticipated 7/06/2024

Actual

Sample size

Target 1000

Update 5224

Reason We added a nested study within the study to assess the effect of different survey advertising methods using fact sheets embedded in consultation software on recruitment rate.

Accrual to date

Update 12

Reason We added a nested study within the study to assess the effect of different survey advertising methods using fact sheets embedded in consultation software on recruitment rate.

Final

Recruitment in Australia

Recruitment state(s) ACT,NSW,NT,QLD,SA,TAS,WA,VIC

Funding & Sponsors

Edit step 8

Funding source category [1] University

Name [1] The University of Sydney, Faculty of Medicine and Health (Next Step Funding)

Address [1] The University of Sydney
NSW 2006
Australia

Country [1] Australia

Primary sponsor type University

Name The University of Sydney

Address The University of Sydney
NSW 2006
Australia

Country Australia

Secondary sponsor category [1] None

Name [1] N/A

**Address [1]**	N/A
**Country [1]**	

Ethics approval

Edit step 9

**Ethics application status**	Approved
**Ethics committee name [1]**	The University of Sydney Human Research Ethics Committee
**Ethics committee address [1]**	The University of Sydney<br>NSW 2006<br>Australia
**Ethics committee country [1]**	Australia
**Date submitted for ethics approval [1]**	
**Approval date [1]**	22/05/2023
**Ethics approval number [1]**	2023/179

Summary

Brief summary

There is no rigorous evidence on the effects of back pain fact sheets among people seeking care for low back pain from their general practitioner. It is uncertain whether a fact sheet resource that focuses more on listing medical management options (JAMA Patient Page- low back pain), can increase a patient's preparedness for decision-making, compared with a resource that focuses on self-management (Australian Commission for Healthcare Safety and Quality Information for Patients Fact Sheet). Our primary aim is to compare preparation for decision-making among people seeking care for low back pain who are given one of two currently available fact sheets. Secondary aims are to examine management intentions, feelings of reassurance about serious pathology and the acceptability of the fact sheets to people seeking care for low back pain.

The study hypothesis is that a fact sheet resource which focuses more on listing medical management options (JAMA Patient Page- low back pain), can increase a patient's preparedness for decision-making more than a fact sheet that focuses on self-management (Australian Commission for Healthcare Safety and Quality Information for Patients Fact Sheet).

Update

There is no rigorous evidence on the effects of back pain fact sheets among people seeking care for low back pain from their general practitioner. It is uncertain whether a fact sheet resource that focuses more on listing medical management options (JAMA Patient Page- low back pain), can increase a patient's preparedness for decision-making, compared with a resource that focuses on self-management (Australian Commission for Healthcare Safety and Quality Information for Patients Fact Sheet). Our primary aim is to compare preparation for decision-making among people seeking care for low back pain who are given one of two currently available fact sheets. Secondary aims are to understand recruitment processes and engagement with research fact sheets, examine management intentions, feelings of reassurance about serious pathology and the acceptability of the fact sheets to people seeking care for low back pain.

The study hypothesis is that a fact sheet resource which focuses more on listing medical management options (JAMA Patient Page- low back pain), can increase a patient's preparedness for decision-making more than a fact sheet that focuses on self-management (Australian Commission for Healthcare Safety and Quality Information for Patients Fact Sheet).

Reason

Added a new method of recuitment

Trial website

Public notes

Private notes

Contacts

Edit step 10

Principal investigator

**Title**	Dr
**Name**	Adrian Traeger
**Address**	The University of Sydney<br>Level 10N, King George V Building, Royal Prince Alfred Hospital (C39)<br>PO Box M179, Missenden Road, NSW, 2050

Country Australia
Phone +61286276231
Fax
Email adrian.traeger@sydney.edu.au

Contact person for public queries

Title Dr
Name Adrian Traeger
Address The University of Sydney
Level 10N, King George V Building, Royal Prince Alfred Hospital (C39)
PO Box M179, Missenden Road, NSW, 2050
Country Australia
Phone +61286276231
Fax
Email adrian.traeger@sydney.edu.au

Contact person for scientific queries

Title Dr
Name Adrian Traeger
Address The University of Sydney
Level 10N, King George V Building, Royal Prince Alfred Hospital (C39)
PO Box M179, Missenden Road, NSW, 2050
Country Australia
Phone +61286276231
Fax
Email adrian.traeger@sydney.edu.au

Data sharing statement

Edit step 11

Will individual participant data (IPD) for this trial be available (including data dictionaries)? No

No/undecided IPD sharing reason/comment: IPD will not be available

What supporting documents are/will be available? No other documents available

Summary results

Edit step 12

No Results

< BACK SUBMIT

You must complete all mandatory fields on step(s) 12 before you are able to submit this trial.

ANZCTR
Home
About us
Statistics
Useful links
News
Contact

Register a trial
Create account
Login
How to register a trial
How to update a trial
Data item definitions
Hints and tips

Search for a trial
Find a trial
How to search
How to get involved

Major funders

Privacy
Terms and conditions
FAQs

Privacy | Disclaimer
Web design by G Squared

Copyright © Australian New Zealand Clinical Trials Registry. All rights reserved.
