# Supplementary material for: One-Page Patient Fact Sheets for Low Back Pain in Primary Care: A Randomized Clinical Trial
Source: JAMA Netw Open. 2025 Jul 17;8(7):e2523352. doi: 10.1001/jamanetworkopen.2025.23352 (PMC12272283; doi:10.1001/jamanetworkopen.2025.23352)
Supplement: Supplement 2. — eMethods. eTable 1. The Intentions to Seek Healthcare eTable 2. Acceptability of the Fact Sheet eTable 3. Characteristics of Completers and Non-completers eTable 4. Results of the Sensitivity Analysis Excluding “Speeders” eTable 5. Results of the Subgroup Analyses eFigure. Flow of Participants for Secondary Outcomes [file jamanetwopen-e2523352-s002.pdf]

Supplementary Online Content

Longtin C, Chang JR, Hersch J, et al. One-page patient fact sheets for low back pain in primary care: a randomized clinical trial. *JAMA Netw Open.* 2025;8(7):e2523352. doi:10.1001/jamanetworkopen.2025.23352

eMethods.

eTable 1. The Intentions to Seek Healthcare

eTable 2. Acceptability of the Fact Sheet

eTable 3. Characteristics of Completers and Non-completers

eTable 4. Results of the Sensitivity Analysis Excluding “Speeders”

eTable 5. Results of the Subgroup Analyses

eFigure. Flow of Participants for Secondary Outcomes

This supplementary material has been provided by the authors to give readers additional information about their work.

eMethods.

After clicking on the link to access the survey and fact sheet, eligible participants were identified via a self-reported screening question (i.e., have you consulted your physician for low back pain in the last 4 weeks?), then received access to the fact sheet. The full PrepDM scale is presented available from https://decisionaid.ohri.ca/eval_prepdm.html.

Categorical variables are presented as frequencies and percentages, and continuous variables as means (SD). Descriptive statistics were used to report participants' sociodemographic and clinical characteristics. Characteristics of non-completers and completers were also described and presented in **Supplement2 eTable 3**.

For secondary outcomes, "healthcare-seeking intentions for low back pain" was defined as a five-category ordinal variable consisting of "definitely take", "likely to take", "unsure", "not likely to take", and "definitely not take". The acceptability of the fact sheet was measured in terms of its length, new information provided, perceived balance between medical and non-medical options presented, comprehensibility, and the likelihood of recommending it to others. Given that responses for length and balance were not ordinal outcomes, we converted five responses into two 3-point ordinal scales: length (appropriate length, slightly long/short, and overly long/short) and balance (balanced, slightly biased, substantially biased). The independent variable was the type of fact sheet, while the covariate was the fact sheet source. Ordinal logistic regression analysis was conducted to examine the effects of fact sheet type on healthcare-seeking intentions and acceptability, controlling for the covariate.

© 2025 Longtin C et al. *JAMA Network Open.*

eTable 1. The Intentions to Seek Healthcare

1. At the moment, which of the following best describes your intentions about **having a scan (x-ray, CT, MRI)** for your low back pain?

Response options:

- o I definitely will have a scan
- o I am likely to have a scan
- o I am unsure
- o I am NOT likely to have a scan
- o I definitely will NOT have a scan

2. At the moment, which of the following best describes your intentions about **taking opioids (strong prescription pain medicines e.g. oxycodone, codeine, tramadol)** for your low back pain?

- o I definitely will take opioids
- o I am likely to take opioids
- o I am unsure
- o I am NOT likely to take opioids
- o I definitely will NOT take opioids

3. At the moment, which of the following best describes your intentions about **using heat (e.g. heat packs, hot water bottle)** for your low back pain?

- o I definitely will use heat
- o I am likely to use heat
- o I am unsure
- o I am NOT likely to use heat
- o I definitely will NOT use heat

4. At the moment, which of the following best describes your intentions about **seeking physical therapy on your back (e.g. massage or spinal manipulation)**?

- o I definitely will seek physical therapy
- o I am likely to seek physical therapy
- o I am unsure
- o I am NOT likely to seek physical therapy
- o I definitely will NOT seek physical therapy

5. At the moment, which of the following best describes your intentions about **staying active** while you have low back pain?

- o I definitely will stay active
- o I am likely to stay active
- o I am unsure
- o I am NOT likely to stay active
- o I definitely will NOT stay active

© 2025 Longtin C et al. *JAMA Network Open.*

eTable 2. Acceptability of the Fact Sheet

1. How would you rate the length of the fact sheet?
- o Much too short
- o A little too short
- o Just about right
- o A little too long
- o Much too long

2. How much of the information in the fact sheet was new to you?
- o All
- o Most
- o Some
- o None

3. The fact sheet talked about different ways to manage back pain. It mentioned **medical care (pain medicines, imaging, surgery)** and **<u>non-medical care</u> (massage, heat, staying active)** for back pain. Did you feel the fact sheet was:
- o Leaning very much towards medical care
- o Leaning a bit towards medical care
- o Balanced
- o Leaning a bit towards non-medical care
- o Leaning very much towards non-medical care

4. How strongly do you agree or disagree with each of the following statements.
- \- I found the information in the fact sheet clear and easy to understand.
- \- I would recommend this fact sheet to other people who are considering their options to treat low back pain.
- o Strongly agree
- o Agree
- o Neither agree nor disagree
- o Disagree
- o Strongly disagree

eTable 3. Characteristics of Completers and Non-completers

Characteristic	All N=1080	Completers N = 803	Non-completers N=277	Standardized difference (95% CI)
Age	(n=1029) 51.9 (14.7)	(n=803) 51.4 (14.9)	(n=226) 53.0 (14.2)	-0.07 (-0.22, 0.07)
Sex	(n=929)	(n=700)	(n=229)	
Female	671 (72%)	515 (74%)	156 (68%)	0.13 (-0.02, 0.28)
Male	254 (27%)	181 (26%)	73 (32%)	-0.13 (-0.28, 0.02)
Prefer not to say	4 (0.4%)	4 (0.6%)	0 (0%)	0.11 (0.01, 021)
Born in Australia (Yes)	(n=1031) 736 (71%)	(n=803) 493 (61%)	(n=228) 157 (69%)	0.02 (-0.13, 0.17)
Non-English spoken at home (Yes)	(n=1031) 182 (18%)	(n=803) 111 (14%)	(n=228) 63 (28%)	-0.28 (-0.43, -0.13)
First episode of low back pain (Yes)	(n=968) 162 (17%)	(n=803) 124 (15%)	(n=165) 28 (17%)	0.14 (-0.02, 0.30)
Pain intensity over the past week[a]	(n=993) 6.8 (1.9)	(n=803) 6.8 (1.8)	(n=190) 6.8 (2.0)	<0.01 (-0.16, 0.16)
Pain intensity categorized[b]	(n=890)	(n=700)	(n=190)	
High Pain	557 (63%)	436 (62%)	121 (64%)	-0.04 (-0.20,0.12)
Low Pain	333 (37%)	264 (38%)	69 (36%)	0.04 (-0.12, 0.20)
Pain duration[c]	(n=915)	(n=700)	(n=215)	
Acute	327 (36%)	263 (38%)	64 (30%)	0.17 (0.01, 0.33)
Chronic	588 (64%)	437 (62%)	151 (70%)	-0.17 (-0.33, -0.01)

Footnote: Continuous variables are presented as mean and standard deviation, while categorical variables are presented as frequencies and percentages. [a]: pain intensity over the past week was assessed by a 0-10 scale (0 = no pain, 10 = worst pain imaginable). [b]: pain intensity was categorized as high pain intensity (> 6/10 on Visual Analogue Scale) vs low pain intensity (≤ 6/10 on Visual Analogue Scale). [c]: pain duration was classified as chronic (> 3 months) vs acute (≤ 3 months) pain.

© 2025 Longtin C et al. *JAMA Network Open.*

eTable 4. Results of the Sensitivity Analysis Excluding "Speeders"

Variable	Adjusted Means (SD)		Adjusted mean difference (95% CI)	Standardized difference (95% CI)	*P* value
	Information sheet N = 313	Advice sheet N = 387			
PrepDM score	59.7 (24.6)	54.8 (24.5)	4.96 (1.26, 8.66)	0.20 (0.05, 0.35)	0.009

Footnote: P-value is from a linear regression model comparing between-group difference, controlling for source of access

© 2025 Longtin C et al. *JAMA Network Open.*

eTable 5. Results of the Subgroup Analyses

Subgroup	Adjusted Means (SD)		Adjusted mean difference (95% CI)	Standardized difference (95% CI)	*P* value
	Information sheet	Advice sheet			
Pain duration*					
Acute	(n=125) 57.8 (26.0)	(n=212) 56.6 (29.2)	1.24 (-4.94, 7.42)	0.04 (-0.18, 0.26)	0.70
Chronic	(n=213) 58.3 (28.9)	(n=224) 51.9 (28.4)	6.39 (1.74, 11.0)	0.22 (0.04, 0.41)	0.007
Pain intensity†					
High pain	(n=257) 57.4 (26.0)	(n=351) 53.6 (26.3)	3.80 (-0.95, 8.56)	0.15 (-0.02, 0.31)	0.11
Low pain	(n=159) 57.7 (28.0)	(n=226) 51.5 (27.9)	6.18 (0.15,12.2)	0.22 (0.01, 0.42)	0.04

Footnote: P-value is from linear regression models comparing between-group difference, controlling for source of access in different subgroups of participants. *: Acute (<3 months) and chronic (>3 months) pain duration. †: High (> 6/10) and low (≤ 6/10) pain intensity thresholds

© 2025 Longtin C et al. *JAMA Network Open.*

eFigure. Flow of Participants for Secondary Outcomes

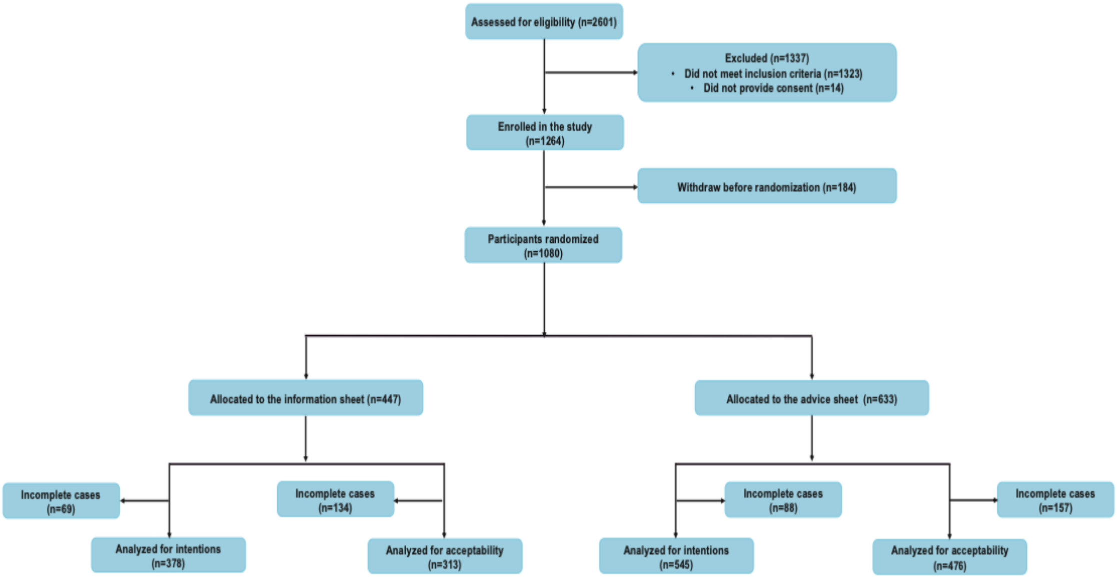

© 2025 Longtin C et al. *JAMA Network Open.*
