# Supplementary material for: One-Page Patient Fact Sheets for Low Back Pain in Primary Care: A Randomized Clinical Trial
Source: JAMA Netw Open. 2025 Jul 17;8(7):e2523352. doi: 10.1001/jamanetworkopen.2025.23352 (PMC12272283; doi:10.1001/jamanetworkopen.2025.23352)
Supplement: Supplement 3. — Data Sharing Statement [file jamanetwopen-e2523352-s003.pdf]

Data Sharing Statement

Longtin. One-Page Patient Fact Sheets for Low Back Pain in Primary Care. *JAMA Netw Open*. Published July 17, 2025. doi:10.1001/jamanetworkopen.2025.23352

Data
Additional Information: Australian New Zealand Clinical Trials Registry (ANZCTR: 12623000603617)
Data available: Yes
Data types: Deidentified participant data
How to access data: Data can be made available on approved requests to the corresponding author.
When available: With publication

Supporting Documents
Document types: None

Additional Information
Who can access the data: Anyone requesting the data with a reasonable justification.
Types of analyses: Purpose for research (e.g., meta-analysis)
Mechanisms of data availability: After approval of a proposal and with a signed data access agreement
